# Deep-sea cabled video-observatory provides insights into the behavior at depth of sub-adult male northern elephant seals, *Mirounga angustirostris*

**DOI:** 10.1371/journal.pone.0308461

**Published:** 2024-09-04

**Authors:** Héloïse Frouin-Mouy, Rodney Rountree, Francis Juanes, Jacopo Aguzzi, Fabio C. De Leo

**Affiliations:** 1 Cooperative Institute for Marine and Atmospheric Studies, University of Miami, Miami, Florida, United States of America; 2 Department of Biology, University of Victoria, Victoria, British Columbia, Canada; 3 The Fish Listener, Waquoit, Massachusetts, United States of America; 4 Instituto de Ciencias del Mar (ICM-CSIC), Barcelona, Spain; 5 Ocean Networks Canada, University of Victoria, Victoria, British Columbia, Canada; University of Ferrara, ITALY

## Abstract

The Ocean Networks Canada (ONC) cabled video-observatory at the Barkley Canyon Node (British Columbia, Canada) was recently the site of a Fish Acoustics and Attraction Experiment (FAAE), from May 21, 2022 to July 16, 2023, combining observations from High-Definition (HD) video, acoustic imaging sonar, and underwater sounds at a depth of 645 m, to examine the effects of light and bait on deep-sea fish and invertebrate behaviors. The unexpected presence of at least eight (six recurrent and two temporary) sub-adult male northern elephant seals (*Mirounga angustirostris*) was reported in 113 and 210 recordings out of 9737 HD and 2805 sonar videos at the site, respectively. Elephant seals were found at the site during seven distinct periods between June 22, 2022 and May 19, 2023. Ethograms provided insights into the seal’s deep-sea resting and foraging strategies, including prey selection. We hypothesized that the ability of elephant seals to perform repeated visits to the same site over long periods (> 10 days) was due to the noise generated by the sonar, suggesting that they learned to use that anthropogenic source as an indicator of food location, also known as the “dinner bell” effect. One interpretation is that elephant seals are attracted to the FAAE site due to the availability of prey and use the infrastructure as a foraging and resting site, but then take advantage of fish disturbance caused by the camera lights to improve foraging success. Our video observations demonstrated that northern elephant seals primarily focused on actively swimming sablefish (*Anoplopoma fimbria*), ignoring stationary or drifting prey. Moreover, we found that elephant seals appear to produce (voluntary or involuntary) infrasonic sounds in a foraging context. This study highlights the utility of designing marine observatories with spatially and temporally cross-referenced data collection from instruments representing multiple modalities of observation.

## Introduction

Understanding the underwater lives of marine mammals is still limited by our inability to fully study the animal’s behavior beneath the water, notably the tactics used by apex predators, such as pinnipeds, to capture their prey. Animal-borne tags have provided an opportunity to gain new insights into the diving and foraging behavior of pinnipeds. In addition to depth, tags can also provide behavioral (e.g., swimming speed) and physiological (e.g., heart rate) information about the animal, and the environment (e.g., light level, temperature, external sound) [1, 2]. However, relatively little is known about how diving and prey characteristics affect the foraging tactics of pinnipeds. Identifying the nature of subsurface behavior among recorded dives often requires many assumptions. To put underwater behavior into context (e.g., foraging event), video systems and data recorders (e.g., time-depth recorder) have been combined, and animal-borne video cameras have been used successfully to document the diet of several pinniped species at sea, such as Weddell seals, *Leptonychotes weddellii* [3, 4], harbor seals, *Phoca vitulina* [5], Antarctic fur seals, *Arctocephalus gazella* [6], Australian fur seals, *Arctocephalus pusillus doriferus* [7], southern, *Mirounga leonina*, [8, 9] and northern, *Mirounga angustirostris*, elephant seals [10, 11].

The northern elephant seal is an apex predator of the North Pacific that alternates terrestrial haul-outs on islands and peninsulas from central Baja California (Mexico) to Oregon (USA) for either molting or breeding with two extended foraging migrations per year [12]. While at sea, northern elephant seals perform routine dive durations of 30 min or more, at common depths of 200–600 m [13, 14], followed by short surface intervals (2–3 min) to reduce predation risk by killer whales and great white sharks while maximizing foraging time [15–19]. Consequently, elephant seals do not rest at the surface, but they rest during deep dives while reducing locomotion cost [20–22]. Recently, Kendall-Bar et al. [23] demonstrated that female elephant seals slept during short naps (less than 20 min) while resting on the ocean floor (64–249 m deep) or drifting through the water column (82–377 m).

Northern elephant seals display intersexual differences in foraging habitat and behavior [12, 24]; males are required to consume more food and consequently, are expected to find prey patches that are denser, larger, or of higher quality than females [25]. Adult male northern elephant seals spend approximately 3–4 months at sea (spring migration) following the breeding season, returning to shore in July/August to molt, while younger males molt from April through June. After approximately one month onshore, adult male northern elephant seals return to sea for 4–5 months (fall migration) before returning to the breeding areas. Males spend less time at sea foraging than females, and they move directly north or northwest to focal foraging areas in the Alaskan Aleutian archipelago’s coastal waters or along the Alaskan and Canadian Pacific coast [12].

Given their large size, long-distance migrations, deep-diving behavior and high fidelity to rookeries [12], elephant seals are an ideal candidate to carry biologging instruments. However, biologging deployments have been heavily biased towards adult female animals due to their higher survival rates, high site fidelity [24], and greater ease of handling [26]. The use of video loggers to estimate diet has been confined exclusively to post-breeding adult females [10, 27, 28]. It is important to note that very little is currently known about where sub-adult male northern elephant seals forage, what they are foraging on and the length of their foraging trips.

Ocean Networks Canada (ONC) maintains large regional cabled observatory networks. The North East Pacific Time-series Undersea Networked Experiments (NEPTUNE) observatory instruments a tectonic plate and overlying ocean at key locations off Canada’s west coast. NEPTUNE is configured with an 800-km network of subsea fiber optic cables connecting multiple seafloor nodes that provide high power and bandwidth for thousands of sensors from the seafloor to the ocean surface. High resolution, continuous, free, open data from these sensors can be accessed via ONC’s digital infrastructure Oceans 3.0 [29]. Its cabled ocean observatories are marine infrastructures equipped with instruments primarily designed to collect data on oceanographic, biogeochemical, and geophysical conditions in real time over long durations [reviewed by 30, 31]. However, most of those nodes are also equipped with High-Definition (HD) video camera and audio equipment, providing unprecedented opportunities to study animal behavior and fishery related dynamics, as well as ecosystem ecology in deep-sea remote environments [30, 32–34], and providing new data for innovative management and conservation strategies [35, 36]. Image collection by ONC observatories provides local information on presence and behavior of marine life, including taxonomic groups such as invertebrates, fish and marine mammals [37, 38].

The Dual-frequency multi-beam IDEntification SONar (DIDSON), acting as an “acoustic camera” has been previously used to monitor marine mammals [39], study deep-diving whales [40, 41], and observe the foraging behavior of a harbor seal in a salmon setnet [42] and a gray seal (*Halichoerus grypus*) in a fish weir [43]. The motivation for the present study was initiated by unexpected observations of elephant seals at 645 m depth during an ongoing Fish Acoustics and Attraction Experiment (FAAE) located at the Barkley Canyon Node, which connects to the main Barkley Canyon observatory study sites. Our study was only made possible due to the innovative use of instruments allowing multiple modalities of observation (simultaneous recordings of underwater sounds, video, and acoustic imaging) in the FAAE project.

## Material and methods

### Ethics statement

Observations of northern elephant seals using cameras did not require approval from an Ethics Committee at the time the data were collected (2022–2023) and did not require permits since the cameras were remotely placed (underwater) and did not cause disturbance to the animals. Data were collected, as part of the FAAE Project, at NEPTUNE’s Barkley Canyon Node, a platform attached to the Neptune observatory that has been already installed since 2009. The Barkley Canyon Node of NEPTUNE is situated outside the boundaries of Marine Protected Areas (MPAs), eliminating the need for special permits from Fisheries and Oceans Canada (DFO). The methodology consisted of recording occurrence of northern elephant seals using HD video and acoustic cameras operating in time-lapse mode to reduce the footprint of exogenous lighting on deep-sea species, as well as a hydrophone for sound detection.

### Study area

**Fig 1:**
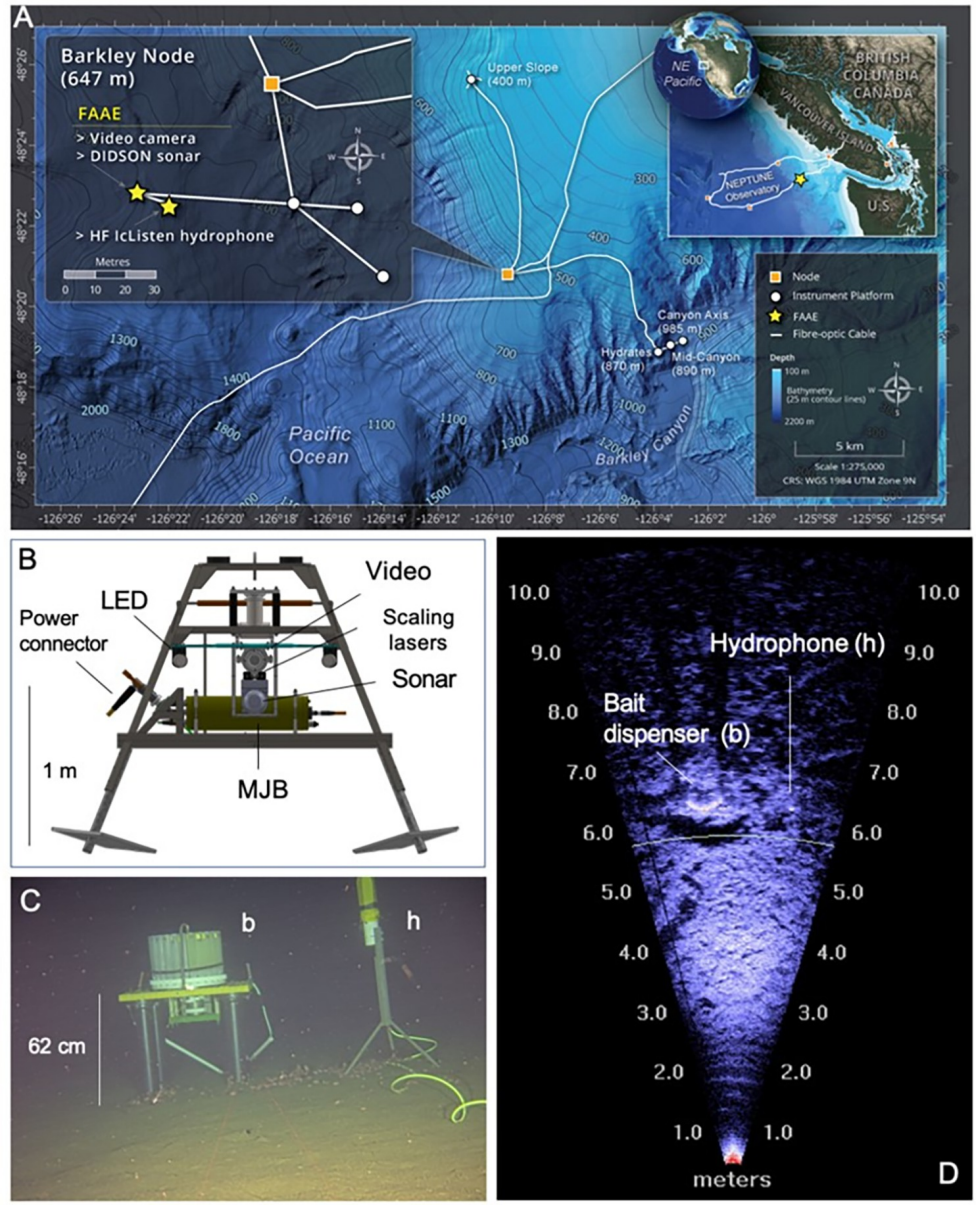
Map of the study area, drawing of the FAAE platform, and field of view for both HD camera and DIDSON sonar. A) Map of the study area with precise location of seafloor instruments and the FAAE platform. Map reprinted from ONC under a CC BY license, with permission from ONC, original copyright 2024. B. Scaled drawing of the FAAE platform with placements of video, sonar, scaling lasers, LED lights, Mini Junction-Box (MJB) housing all device controlling electronics; and the connector plug where fiber-optic and power are exchanged with the main instrument platform. C. Field of view of the camera showing the bait dispenser (b) and hydrophone (h) in frame. D. DIDSON sonar field of view cone (14° deg. horizontal) showing the backscatter signal and the positions of the bait dispenser and hydrophone.

The study site (48°34’57.9"N, 126°15’77.1"W) is located ~100 Km offshore from Barkley Sound, Vancouver Island, NE Pacific, at a depth of 645 m (Fig 1A). The NEPTUNE’s Barkley Canyon Node observatory location is connected by a fiber-optic cable which also supplies power (10,000 VDC) from the shore station in Port Alberni, BC, and branches out to feed three other study locations inside Barkley Canyon (870–970 m) and one at Barkley Upper Slope (400 m) [44; Fig 1A]. Like all NEPTUNE’s subsea infrastructure, Barkley Node hosts a suite of biogeochemical and oceanographic sensors with data flowing in real-time to the shore station, and then archived at the University of Victoria’s servers. All data are public and freely accessible through ONC’s Oceans 3.0 data management system (https://data.oceannetworks.ca/home).

Barkley Canyon incises the continental shelf near the 200 m isobath at its head and reaches ~2,200 m at its mouth providing a direct conduit between the continental margin and the Cascadia Basin [33, 45]. Summer upwelling-favourable winds promote stronger up-canyon current flows, allowing for deep, cold, nutrient rich and low oxygen waters to move onto the slope and shelf, and closer to the surface, enhancing primary productivity [46]. During winter, when downwelling winds are dominant, deep wind-driven mixing forces oxygen-rich surface waters down onto the slope. Short-lived phytoplankton blooms also occur during winter/downwelling conditions, and rapidly export particulate carbon through the main canyon axis [47]. Due to enhanced primary productivity and pelagic-benthic coupling near Barkley Canyon and adjacent slope, the seafloor harbors high benthic abundance and biomass [48, 49]. Benthic habitats combine methane seeps with hydrate outcrops, patches of solitary black corals and flat soft-sedimented seafloor, all bathed in hypoxic waters within upper and lower boundaries of the NE Pacific oxygen minimum zone [49–52].

### Experimental set up

Barkley Canyon Node was the selected site for the installation of a FAAE designed to combine observations from video, acoustic imaging sonar, and underwater sounds from a hydrophone, to examine the effects of light, bait introduction and background noise on fish and invertebrate behaviors (Fig 1B and 1C and S1 Fig). To date the project has included two operational phases: a pilot stage (FAAE-I) from September 17, 2019 to February 3, 2020 and full-scale phase (FAAE- II) from May 21, 2022 to July 16, 2023. The FAAE-II platform contained a HD color Axis P1378 Sony video camera (1080p), two ROS LED lights (Remote Ocean Systems, 100 W, >406 lumen), a pair of scaling lasers (10 cm apart) and a DIDSON sonar (DIDSON-3000, SoundMetrics) (Fig 1B). The sonar was used to examine the effect of artificial lighting on benthic species behaviors. The HD video camera was centered on the FAAE platform, just atop of the sonar transducer head, to optimize the overlapping of Fields Of View (FOV, Fig 1B) and allow comparison of faunal detections between the instruments. After placement of the platform, the final sonar FOV recording was set as follows: approximately 14° vertical, 29° horizontal and height above the seabed of 47.4 cm (imaged area ~25 m^2^), while the HD camera FOV recording was set as follows: 19.5° vertical, 34° horizontal and height above the seabed of 63 cm (imaged area ~12 m^2^).

To examine the soundscape, and any potential influence of biological or anthropogenic sounds at the site, a 16-bit Ocean Sonics icListen HF Hydrophone (Ocean Sonics Ltd), cabled and plugged into the FAAE platform, was placed ~ 6 m from the center of the video camera and sonar transducer head (Fig 1C and 1D and S1 Fig). Finally, the FAAE experiment set up also included an autonomous bait release system, which consisted of a TechniCap sediment trap carousel mounted upside down in a 62 cm tall 4-legged high-density PVC mount (Fig 1C and 1D and S1 Fig). Each of the 24 bottles in the carousel were pre-loaded with a single ~300 g sardine affixed to a 30-cm 1/4” steel rod and filled with cooking vegetable oil. The carousel was pre-programmed to release one sardine approximately at 19:12 (Universal Time Coordinated, UTC) every 14 days from May 30, 2022 to May 1, 2023. The 14-day intervals and use of cooking oil aimed at maximizing bait effectiveness in attracting scavengers by delaying spoiling [53]. Bait release times were synchronized so they would be captured during video recording.

The entire sampling routine of the FAAE platform is summarized in S1 Fig. In short, while the hydrophone continuously recorded at a 128 kHz sampling rate, hourly the sonar recorded for 15 minutes at 3 frames per second (fps) and the video camera at 30 fps for 4 minutes and 40 seconds. Typically, all video recordings from NEPTUNE observatory cameras are limited to 5 minutes or less, in an effort to reduce faunal disturbance from the artificial lighting [33, 54, 55]. The video recording, triggered by the LED lights turning on, started at elapsed 5 minutes from the start of the sonar recording, and ended approximately 5 minutes before the end of the sonar recording (S1C Fig). This routine allowed for ~5 minutes of sonar-only recording before light disturbance, ~5 minutes of overlapping recording, and ~5 minutes of sonar-only recording post light disturbance (S1C Fig). The HD video camera recorded for ~2.5 minutes at its widest zoom setting, aiding an illuminated FOV of ~12 m^2^; and for ~2.5 minutes zoomed in and focused on the bait release carousel, with an FOV of ~6 m^2^ (S1B Fig). On September 19, 2022, the sonar recording routine was split into two different FOVs (S2 Fig). At bi-hourly intervals it recorded using 5 m and 10 m as the window-length (maximum range). If not mentioned otherwise, all results discussing time are presented in Local Standard Time (LST; UTC-8h).

### Data analysis

Video, audio and acoustic imaging data from FAAE-II were accessed via ONC’s web-based video library tool, SeaTube Pro (https://data.oceannetworks.ca/SeaTube), from the Oceans 3.0 data archiving system suite [29, 56]. We analyzed observatory time-series data from FAAE-II from May 21, 2022 (deployment date) to July 16, 2023 (retrieval date), for a total of 422 days, to assess the occurrence and behavior of elephant seals at this site.

All available imagery from the fixed seafloor HD camera was reviewed to determine daily and hourly presence of elephant seals at Barkley Canyon Node. We found elephant seal events distributed in seven periods (P1 to P7, Table 1, S2 Fig), which ranged from June 22 to July 7, 2022 (16 days, P1), from July 23 to August 8, 2022 (17 days, P2), from August 26 to September 8, 2022 (14 days, P3), from September 30 to October 18, 2022 (19 days, P4), from October 29 to November 8, 2022 (11 days, P5), from December 4, 2022 to January 26, 2023 (54 days, P6) and from May 14 to May 19, 2023 (6 days, P7). Due to time constraints, only sonar videos corresponding to each period (in addition of 24h before and after) as well as all sonar videos before the first elephant seal sighting and from the last elephant seal sighting (November 8, 2022) to 11 November 2022 (when the DIDSON failed), were also reviewed for individual presence. Because P4 and P5 were so close, sonar videos between both periods were also reviewed for elephant seal presence.

**Table 1:** Elephant seal presence at Barkley Canyon Node. Details provided for each period (dates range, total events as hourly bins, total events on the HD videos and total events on the sonar videos) and for each identified elephant seal (age class, dates range of presence, timespan, total events and count of different days for which each elephant seal has been observed).

**Period**	**P1**	**P2**	**P3**	**P4**		**P5**	**P6**		**P7**
**Dates range**	Jun 22 –Jul 7, 2022	Jul 23 –Aug 8, 2022	Aug 26 –Sep 8, 2022	Sep 30 –Oct 18, 2022		Oct 29 –Nov 8, 2022	Dec 4, 2022 –Jan 26, 2023		May 14 –May 19, 2023
**# Total events (hourly bins)**	26	30	32	105		27	12		12
**# Events on the HD videos**	16	13	18	37		5	12		12
**# Events on the sonar videos**	24	28	27	104		27	0 (failed)		0 (failed)
***Identified ES (age class)***	***Brian (SA1)***	***Dennis (SA3)***	***Carl (SA3)***	***Al (SA3)***	***Mike (SA4)***		***David (SA3)***	***Blondie (SA3)***	***Bruce (SA4)***
***Presence—Dates range***	*Jun 22 –Jul 6*, *2022*	*Jul 23 –Aug 2*, *2022*	*Aug 26 –Sep 8*, *2022*	*9 Oct*, *2022*	*Sep 30 –Oct 29*, *2022*		*4 Dec*, *2022*	*Dec 16–18*, *2022*	*May 14–17*, *2023*
***timespan***	*15 days*	*11 days*	*14 days*	*1 day*	*30 days*		*1 day*	*3 days*	*4 days*
***# events***	*11*	*8*	*7*	*1*	*20*		*1*	*2*	*8*
***# different days***	*9*	*5*	*6*	*1*	*9*		*1*	*2*	*4*

Video files were reviewed with Behavioral Observation Research Interactive Software [BORIS; 57] to both analyze the seal’s individual behavior and the presence of potential prey. This open-source software allows the user to set up an ethogram as a list of different behavioral events such as active foraging event, resting event (lying down or staying immobile), swimming event, visible potential prey presence (S3 Fig), and other notable behaviors. Then the program records the timing of those behavioral events. Because it was more difficult to discern specific behaviors on the sonar videos, a simpler analysis was performed on them. In each sonar video, the first and last appearance of the elephant seal was noted. The variation in presence duration (between the first and last appearances) was compared between the HD and sonar videos using a Wilcoxon test. The variation in the first appearance (in seconds; from the start of the video) of elephant seals on the HD camera was compared between periods when the sonar was functional and periods when the sonar failed using a Wilcoxon test. To estimate potential influence of the artificial LED lights on elephant seal behavior, their behavior states were noted in sonar videos for each period: before HD video recording (lights off), during HD video recording (lights on) and after HD video recording (lights off). Each period was approximately 5–min long and behaviors included: resting (lying down or staying immobile), passing through (swimming) or being absent (if a resting animal was suddenly swimming away when lights turned on, it was counted as absent in the period corresponding to the HD video camera recording). The occurrence of potential fish, cephalopod and crab prey for the elephant seals was noted for each HD video in which elephant seals were observed (other potential prey such as shrimp were often abundant, but not quantified).

In order to investigate diel (i.e. 24-h based) occurrence, each hour-bin with elephant seal presence was noted. A chi-square goodness-of-fit analysis was used to determine whether the observed frequency distribution of the number of elephant seal events in each hour bin differed significantly (α = 0.05) from the expected distribution.

Individual elephant seals were identified, when possible, based on visible body marks or scars, as well as the eyeliner cues (S4 Fig). These markings near the eyes accumulate while elephant seals are at sea (possibly due to a diatom growth; Colleen Reichmuth, personal communication) and consequently, were used only for each period and not across periods due to possible change across longer time intervals. Each individual was further identified by sex/age class category based on physical features (i.e., size and proboscis development) following descriptions provided in Casey et al. [58] and detailed in S1 Table. Because the proboscis, as well as the body, present some compression at that depth (645 m) it was important to rely more on the chest shield development to age male elephant seals (Colleen Reichmuth, personal communication). Following adolescence (ages 1–3 years), male elephant seals accelerate through rapid reproductive development over a 4-year period (sub-adult, ages 4–8 years), during which they more than triple in body size [59].

Sound files, encompassing the 15-min sampling window around each video in which an elephant seal was observed, were downloaded from Sea Tube Pro, amplified by 30 dB prior to analysis, and examined for potential elephant seal and fish sounds related to observed behaviors. All processing was conducted in Raven Pro Interactive Sound Analysis Software version 1.5 (Cornell Lab of Ornithology, Ithaca, NY, USA). Spectrograms and waveforms of sounds were visually and aurally examined to identify any sound of potential interest (Spectrograms with 1,024-sample FFT, Hanning window, 50% overlap). Sounds were then approximately synchronized with and embedded within the video based on file time stamps. ONC uses an internal master clock that all observatory instruments are synchronized to. Loud sounds observed when sablefish (*Anoplopoma fimbria*) bumped into the instruments were then used to adjust the synchronization to allow precise comparison of sound events with behavioral observations. Sound and video recordings were first processed separately and then iteratively reviewed. Events first identified in the audio processing were examined in the video, and events identified in the video (e.g., elephant seal attack on a fish) were examined in the spectrogram to identify sounds and associated behavior whenever possible. Power spectra of select sounds were analyzed in SpectraPro332 (Sound Technology, Inc.).

## Results

In the 9737 reviewed ~5-min clips (~811h) from the video camera, elephant seals were observed in 113 videos. In the 2805 reviewed ~15 min acoustic video clips (~701 h) from the DIDSON sonar, elephant seals were observed in 210 recordings. Elephant seals were first observed on June 22, 2022 on both instruments, but were last observed on November 8, 2022 on the sonar videos, and on May 19, 2023 on the HD videos. By combining both datasets, elephant seals were observed in 244 hourly bins, 34 only in HD videos, 79 in both HD and sonar videos, and 131 only in sonar videos. Elephant seal occurrences were distributed as: 26 events (16 events on the HD videos and 24 events on the sonar videos) in P1 (June 22, 2022 –July 7, 2022), 30 events (13 events on the HD videos and 28 events on the sonar videos) in P2 (July 23, 2022 –August 8, 2022), 32 events (18 events on the HD videos and 27 events on the sonar videos) in P3 (August 26, 2022 –September 8, 2022), 105 events (37 events on the HD videos and 104 events on the sonar videos) in P4 (September 30, 2022– October 18, 2022), 27 events (5 events on the HD videos and 27 events on the sonar videos) in P5 (October 29, 2022 –November 8, 2022), 12 events only on the HD videos (DIDSON failed) in P6 (December 4, 2022 –January 26, 2023) and 12 events only on the HD videos (DIDSON failed) in P7 (May 14, 2023 –May 19, 2023).

### Video vs acoustic imagery

**Fig 2:**
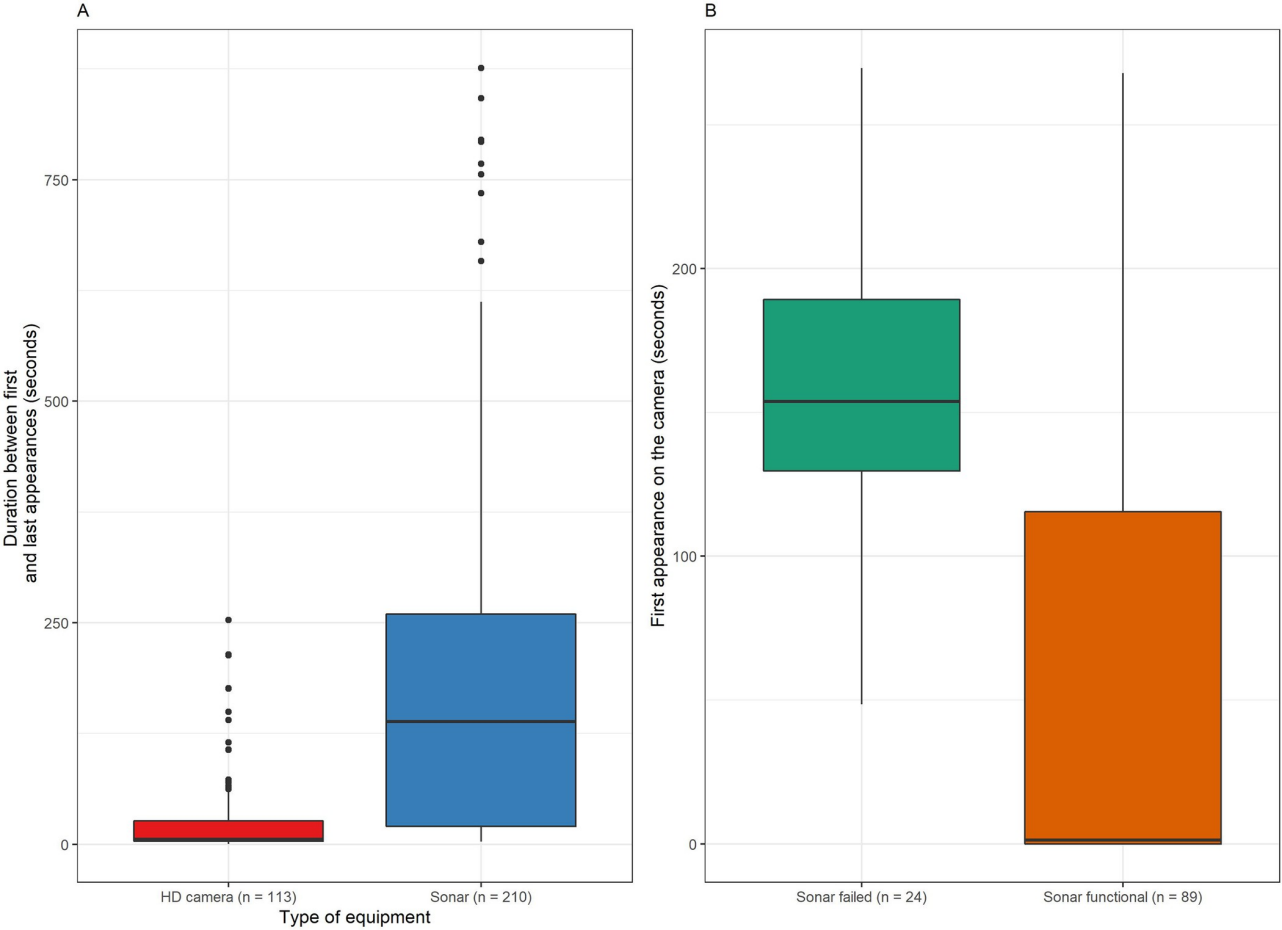
Elephant seal presence duration and first appearance. A) Box plot of elephant seal presence duration (in seconds) between the first and last appearances on HD camera (red) and sonar (blue) videos. Sonar videos (n = 210) are approximately 15 min long each. Camera videos (n = 113) are approximately 4 min 40 sec long each. B) Box plot of first appearance (in seconds) of the elephant seals on the HD videos for periods when the sonar had failed (green, n = 24) or was functional (orange, n = 89). Middle line shows the median, while lower and upper lines of the boxes show 25th and 75th percentiles, respectively. The ends of the vertical line indicate minimum and maximum values and the dots indicate outliers.

Scanning distance on the sonar camera was not consistent through the study period and might have limited our ability to detect elephant seal presence if the animal was further than ~10 m (Fig 1 and S2 Fig). In 90% of instances when an elephant seal was found on HD but not on sonar videos, the elephant seal was found swimming in the background, at least 8 m away from the sonar. In the last instance, the animal was in front of the sonar but not near the seafloor. To be detected by the sonar recordings, elephant seals need to be in the FOV and near to the bottom. Similarly, recording duration on the HD video camera varied through the study period and on rare occasions might have limited our ability to identify elephant seal individuals or better define behavior at depth (S2 Fig). Total duration of presence for each elephant seal event was estimated using the time difference between the first and last appearance of the animal in each video (HD videos are approximately 5 min long and sonar videos are approximately 15 min long). A large, significant discrepancy in seal presence was observed between video types (W = 3617, p < 0.001; Fig 2A). Elephant seals were found for longer periods on sonar videos (median = 139 s) compared to HD videos (median = 5.6 s). The timing of the first appearance of elephant seal on the HD camera was significantly different between periods when the sonar was functional and periods when the sonar had failed (W = 1754, p < 0.001; Fig 2B). Elephant seals were observed earlier on the HD camera for periods when the sonar was functional (median = 1.3 s) compared to periods when the sonar had failed (median = 154 s).

**Fig 3:**
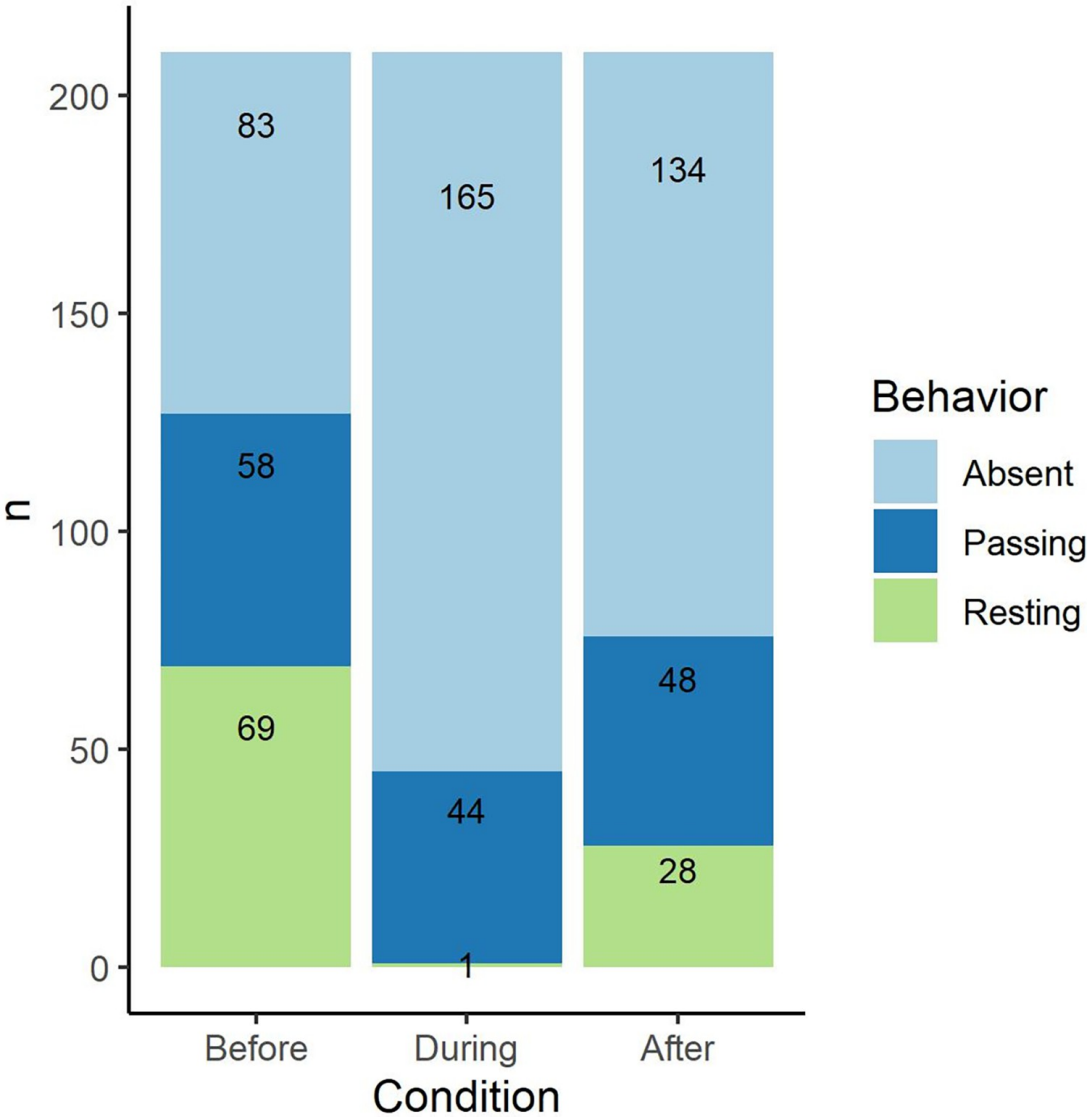
Elephant seal behavior for each period. Stacked bar chart illustrating for the 210 sonar videos with elephant seal presence, the count (# for each behavior) of each behavior relative to each period (Before: Before lights turn on; During: When lights are turned on and HD camera is recording; After: After lights are turned off). Each period is approximately 5 min long. Behaviors included: Resting (lying down or staying immobile), Passing through (swimming) or Absent (if a resting animal was suddenly swimming away when lights turned on and did not come back later it was counted as absent in the period corresponding to the HD camera recording).

On the sonar recordings, before lights were turned on, elephant seals when present (127/210 events; 60%) were mostly resting (69/210 events; 33%; Fig 3). When lights turned on and HD video started recording, most of the elephant seals present during the first period left, and the ones still present or coming in the FOV were only passing through (44/210 events; 21%), and only one still remained resting for a few minutes (1/210 events). After the lights turned off and the HD video stopped recording, elephant seals appeared, came back, or continued to stay in the sonar FOV (present 76/210 events; 36%), with a small proportion of elephant seals resuming or starting a resting activity (28/210 events; 13%). Of the 69 resting elephant seals in the “Before” category, 59 left when the lights turned on, 9 were still present but passing and 1 continued its rest (then left before lights turned off). Of the 59 elephant seals that left when the lights turned on, 6 came back after the lights turned off, 3 were observed swimming and 3 resumed their rest. Of the 9 elephant seals that were still present and swimming when lights turned on, 6 were absent when lights turned off, while 2 were still swimming, and 1 resumed its rest.

### Elephant seal occurrence at Barkley Canyon deep-sea node

**Fig 4:**
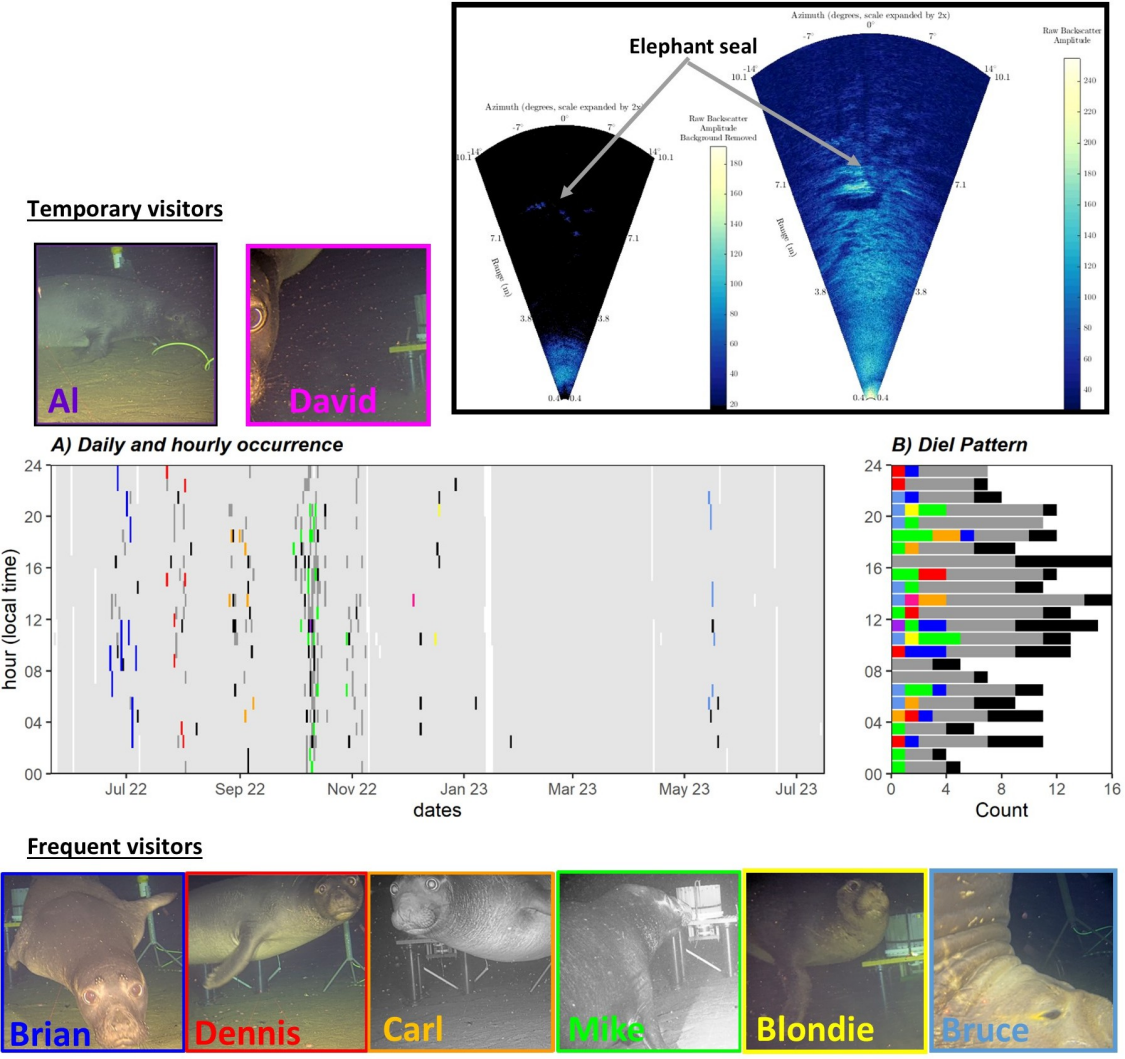
Occurrence of individual elephant seals at Barkley Canyon Node. A) Diel and hourly occurrence of elephant seals at Barkley Canyon Node in 1-h bins. Light grey areas indicate that either the DIDSON sonar or the video camera were recording, or both. Dark grey shaded areas indicate elephant seal presence only in the sonar (example at the top). Black areas indicate unidentified seals present on video recordings only or on both video and sonar recordings. Colored areas indicate identified individuals (Brian, Dennis, Carl, Mike, Al, David, Blondie and Bruce) on the video camera. White areas indicate a lack of any recordings. Photos reprinted from ONC under a CC BY license, with permission from ONC, original copyright 2024. B) Diel pattern of elephant seal presence as individuals (colored bars), unidentified (black bars) on the video camera or found only on the sonar (dark gray bars), expressed in local time (UTC– 8hr).

In both camera and sonar videos, elephant seals were present as single individuals and were never observed in a group. Eight individual elephant seals, all males, were identified in the period June 2022 –May 2023 (P1-P7, S2 Fig, Fig 4 and Table 1) and were named as follows: Brian (SA1; estimated 4 years old), Dennis (SA3; estimated 6 years old), Carl (SA3; estimated 6 years old), Mike (SA4; estimated 7 years old), Al (SA3; estimated 6 years old), Blondie (SA3; estimated 6 years old), David (SA3; estimated 6 years old) and Bruce (SA4; estimated 7 years old). Although elephant seals at several events could not be identified on the HD videos, it seemed that each period (P1 –P7) corresponded mainly to one individual (Table 1). During P1, Brian frequently visited the site for a period of 15 days (identified 11 times on 9 different days from June 22 to July 6, 2022). During P2, Dennis frequently visited the site for a period of 11 days (identified 8 times on 5 different days from July 23 to August 2, 2022). During P3, Carl frequently visited the site for a period of 14 days (identified 7 times on 6 different days from August 26 to September 8, 2022). During P4 and P5, Mike frequently visited the site for a period of 30 days (identified 20 times on 9 different days from September 30 to October 29, 2022). Both Al and David were found only once, during P4 (on October 9, 2022) and P6 (on December 4, 2022), respectively. During P6, Blondie was found twice on a period of 3 days (on December 16 and 18, 2022). During P7, Bruce frequently visited the site on a period of 4 days (identified 8 times on 4 consecutive days from May 14 to 17, 2023). Elephant seals seemed to visit the site more frequently during daylight hours (10 am to 9 pm; Fig 4) though this trend was not significant (Chi-square test, χ2 = 24.09, p = 0.4, df = 23). Regarding the timing of the elephant seal presence, there might be some relationship with moon and tidal phases in P1 –P3, but this relationship was absent in P4 –P7 (S5A and S5B Fig). The oxygen concentration was low (approximately 0.1–0.6 ml/l; corrected) during all the study period (S5C Fig). Interestingly, an abrupt reduction in dissolved oxygen took place during P4 concurrently with the departure of elephant seals. Elephant seals came back during P5 when dissolved oxygen levels increased (S5C Fig). Elephant seals were not present during the same time period, or shortly thereafter, when the bait release occurred, except for one isolated occasion (S5D Fig).

### Elephant seal behavior

**Fig 5:**
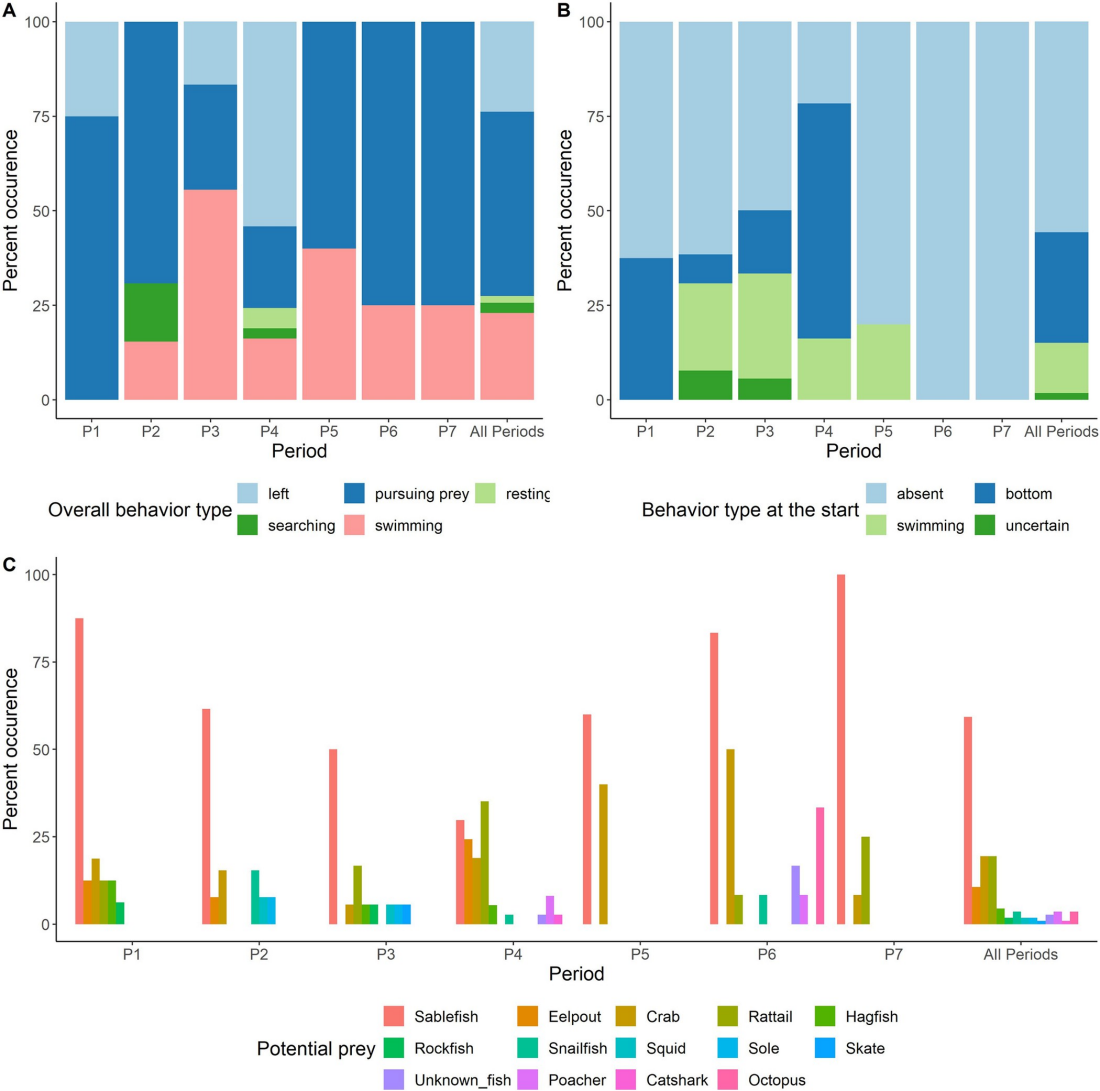
Elephant seal behavior types and possible prey types observed in HD videos. Percent occurrence of elephant seal behavior types within 113 HD videos where elephant seals were observed A) overall behavior in the video; B) behavior observed at the start of the video recording; C) possible prey types observed in the same video as the elephant seal (not necessarily at the same time).

Of the 113 HD videos in which elephant seals were observed, actively pursuing prey was the most frequent behavior (N = 55, 49%), followed by immediate departure (N = 27, 24%), swimming through (N = 26, 23%), searching (N = 3, 2%), and resting (N = 2, 2%, Fig 5A). One elephant seal took a few seconds to move away at the start of the video and thus, it was annotated as resting but not immediate departure. Elephant seals were mostly absent at the start of the video (N = 63, 56%). Otherwise, they were usually sitting on the sea floor or just lifting off (N = 33, 29%), or swimming past (N = 15, 13%, Fig 5B). Differences in behavior among the elephant seals were noted (Fig 5A). Elephant seals in P1, P2, P5, P6 and P7 were most likely to be actively pursuing prey. Elephant seals in P3 were most likely to be observed swimming through, and in P4 were most likely to immediately leave the area based on the HD video. Actively pursuing prey most often involved a slow chase where the elephant seal followed along a sablefish flight path from above (N = 31; example in S8 Video) and as much as 3 s behind. Close chases (N = 8; example in S3 Video) and flanking chases (N = 2; example in S4 Video) were less common. A direct vertical chase from above was observed once when an elephant seal followed a fast-darting unidentified prey (likely a squid, in S2 Video) which crashed into the sea floor. Elephant seals oriented their head towards moving fish targets (example in S7 Video). Moreover, prolonged whiskers protractions were observed on elephant seals (e.g., Fig 4, Brian) when prey were present. The elephant seals seem to primarily hunt moving prey, and mostly ignore stationary or drifting prey (see below). On 11 occasions in 7 videos elephant seals were observed bobbing their heads from 2 to 10 times on close approach to a sablefish (S2 Table). A low frequency (infrasonic) acoustic signal was recorded concurrently with head bobbing (see below).

### Prey selection of the elephant seal

**Fig 6:**
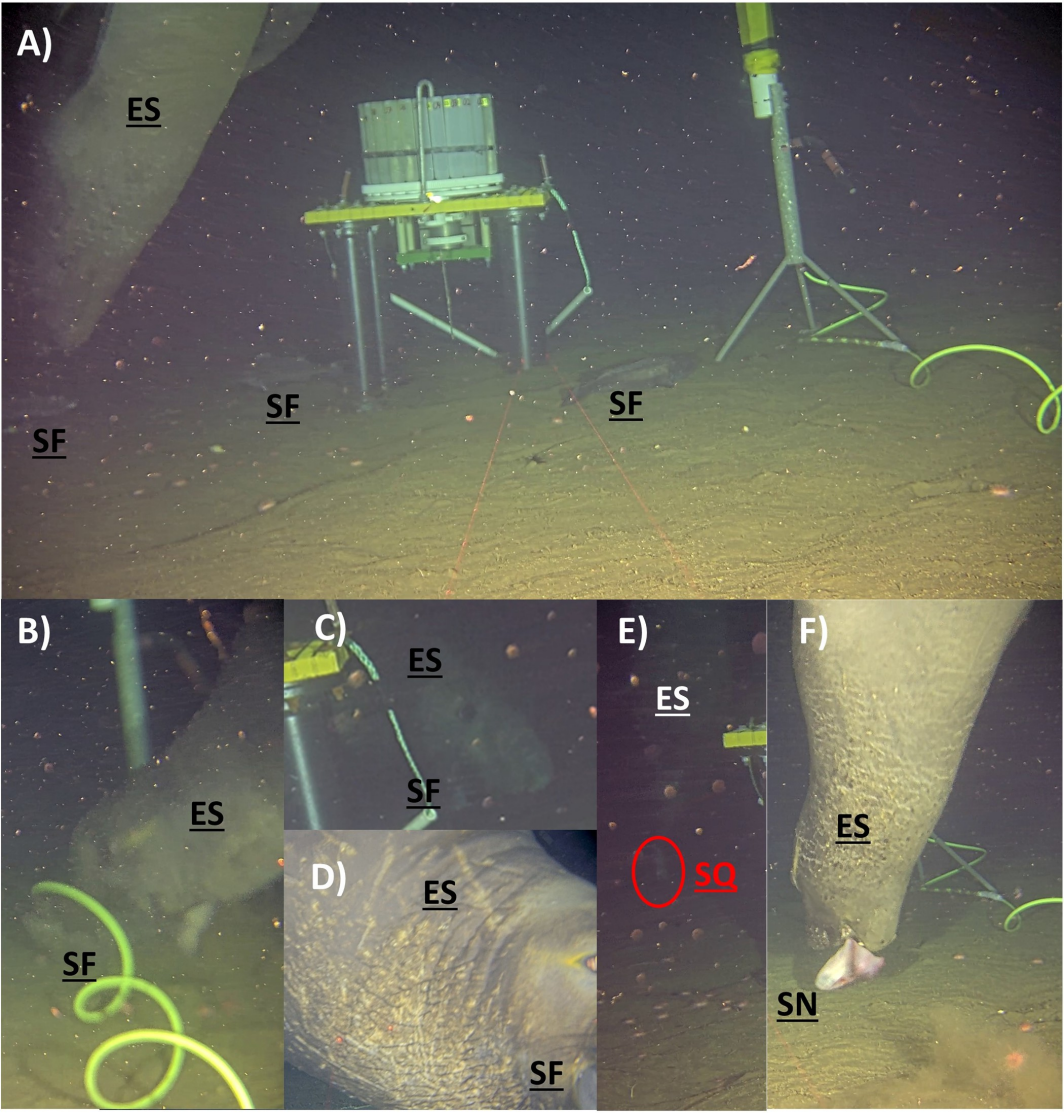
Foraging behavior and diet of elephant seal. A) On July 27, 2022 one individual (Dennis) elephant seal (ES) actively hunted the two swimming sablefishes (SF, on the left) but ignored the immobile sablefish (SF, in the center). B) On August 2, 2022 one individual (Dennis) elephant seal (ES) successfully caught one sablefish (SF). On both May 16 (C) and May 17 (D), 2023 one individual (Bruce) elephant seal (ES) successfully caught one sablefish (SF). E) On August 5, 2022, in the background on the video, one individual elephant seal (ES) pursued and apparently successfully caught that seemed a squid (SQ, based on the propulsive swim). F) On July 23, 2022 one individual (Dennis) elephant seal (ES) mistakenly caught a snailfish (SN) in an attempt to capture a sablefish. The snailfish was rapidly released. [Videos for events A–F are in Supplemental materials]. Photos reprinted from ONC under a CC BY license, with permission from ONC, original copyright 2024.

Fourteen potential prey types were identified in the video recordings when elephant seals were present (Fig 5C and S3 Fig). Those potential prey types, which were also the most common species observed at the site, included sablefish (Anoplopomatidae, 58%), crabs (combined tanner *Chionoecetes* spp., Oregoniidae and Scarlet king crabs *Lithodes couesi*, Lithodidae, 20%), rattails (Macrouridae, 20%), eelpouts (Zoaracidae, 11%), Pacific hagfish (*Eptatretus stoutii*, Myxinidae, 4%), snailfish (*Careproctus* sp., Liparidae, 4%), blackfin poacher (*Bathyagonus nigripinnis*, Agonidae, 4%), and giant Pacific octopus (*Enteroctopus dofleini*, Enteroctopodidae, 4%). Rockfish (Scorpaenidae), unidentified squid (Cephalopoda), Dover sole (*Microstomus pacificus*, Pleuronectidae), longnose skate (*Caliraja rhina*, Rajidae), brown catshark (*Apristurus brunneus*, Pentanchidae), and unidentified deep-sea fish, each occurred in less than 3% of the videos when elephant seals were present. At least one potential prey species was observed in 84% of the videos with elephant seals. However, many of those were small rattails or eelpouts. When considering only prey large enough to be of potential interest to the elephant seal, the potential prey availability dropped to 66% (Fig 5C). Based on HD videos, the most abundant species, sablefish, was the preferred prey targeted by elephant seals (Figs 5C, and 6A–6D). Of the 55 foraging events, four were successful feeding attempts, three on a sablefish (S1, S5 and S6 Videos) and one of an unknown prey, likely a squid, observed in the background (S2 Video, Fig 6E). A fourth sablefish may have been caught just off-screen based on struggle sounds (S3 Video). In another case an elephant seal descending from above startled a sablefish resting beneath the bait dispenser carousel and immediately gave chase (S8 Video). Sablefish were most likely to be present in videos during P1 (88%, Fig 5C), P6 (83%, Fig 5C) and P7 (100%) but least likely to be present in P4 (30%) which corresponded to a low level of dissolved oxygen (S5 Fig). However, the highest diversity of potential prey occurred also during P4. Interestingly, elephant seal appeared to actively avoid or ignored a snailfish in three of four videos where they occurred together. In one video (S7 Video), an elephant seal (Dennis) inspected then ignored a moving snailfish and later, when mistakenly caught during an attempt to capture a sablefish (Fig 6F), the elephant seal quickly released it. In another video (not provided) an actively moving snailfish was ignored despite proximity. However, in that case the elephant seal was closely pursuing an evasive sablefish.

### Time spent at depth and resting activity

Many of the elephant seal detections in the HD videos were of brief appearances (26.6 ± 4.90 s). However, based on the sonar videos (~15 min or 900 s long), elephant seals were present on the sea bottom for at least 178.3 ± 13.2 s (3–876 s) (mean ± standard error; minimum–maximum) with a combination of laying at the bottom (for several seconds) or swimming in the area. As discussed previously, elephant seals were observed much more frequently on the sonar videos and before the HD camera was turned on, staying immobile until the pair of LED lights were turned on, which triggered a strong and immediate behavioral reaction in elephant seals (S9 Video). On one occasion, an elephant seal remained motionless between the instruments, facing away from camera, for 8 s before strongly reacting (S9 Video). On another occasion, elephant seal’s rest was not interrupted by the LED lights turning on, due to the body orientation (S9 Video) and the animal remained motionless for a total of 520 s (8 min 40 s, S18 Video). The actual duration of the rest might have been longer than 520 s because the animal was already lying down when the sonar started recording.

### Sound occurrence

**Fig 7:**
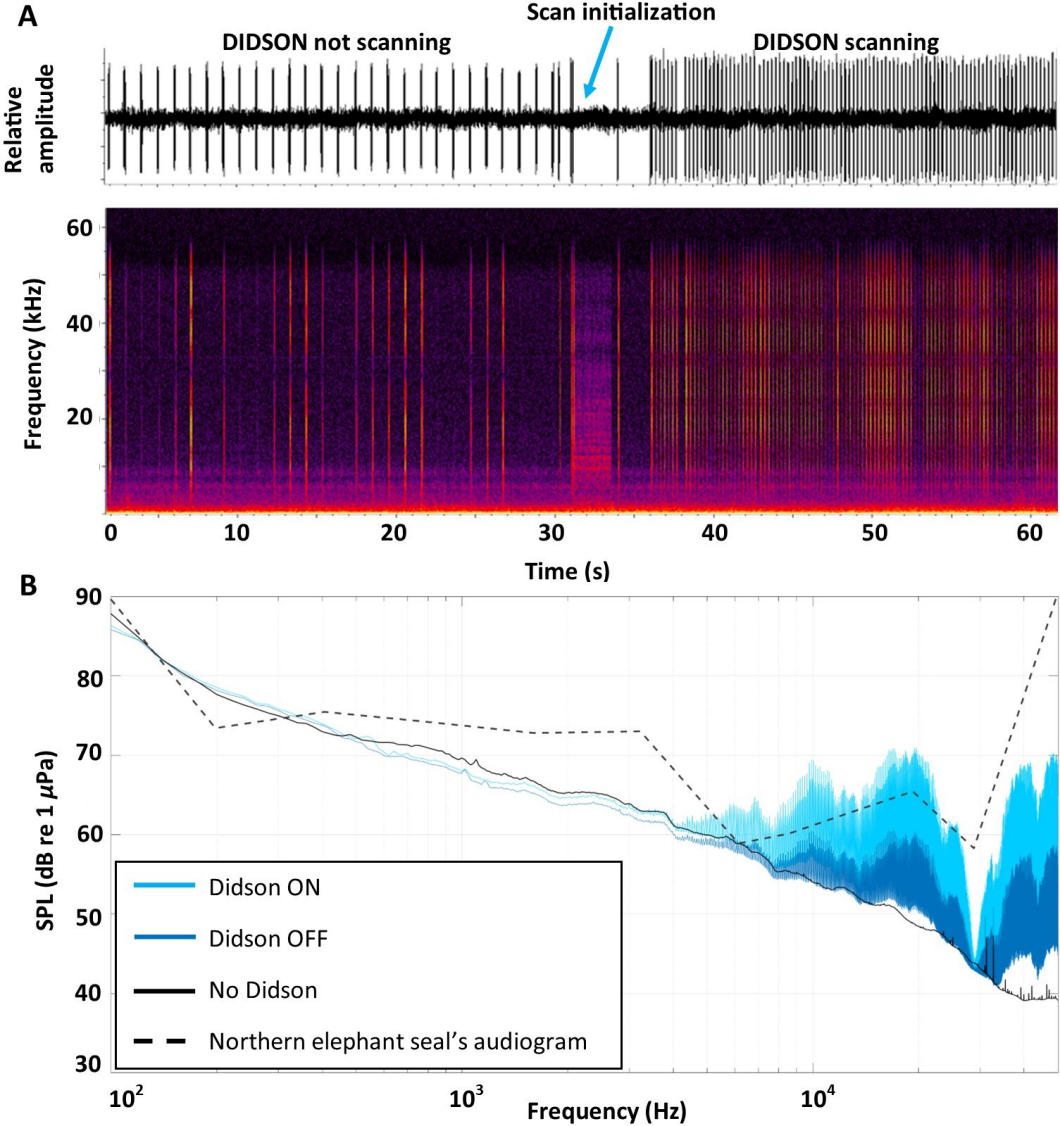
Sound produced by the sonar and northern elephant seal hearing range. A) Waveform (top) and spectrogram (bottom) of sound production by the DIDSON acoustic camera as it transitioned from continuous pings approximately every second produced in the standby mode, to the more rapid ping rate of about 4 pings/s while in recording mode. Spectrogram parameters: 1024 point Hanning windowed FFT with 50% overlap. B) Spectrum density level for periods when the Didson was turned ON (scanning mode; 15 min every hour) on 10 June 2022 (light blue line), when the Didson was turned OFF (standby mode; 45 min every hour) on 10 June 2022 (dark blue line), and without the Didson on 10 June 2023 (black line). The dashed line is the estimated northern elephant seal audiogram based on behavioral threshold data from Kastak and Schusterman [60].

**Fig 8:**
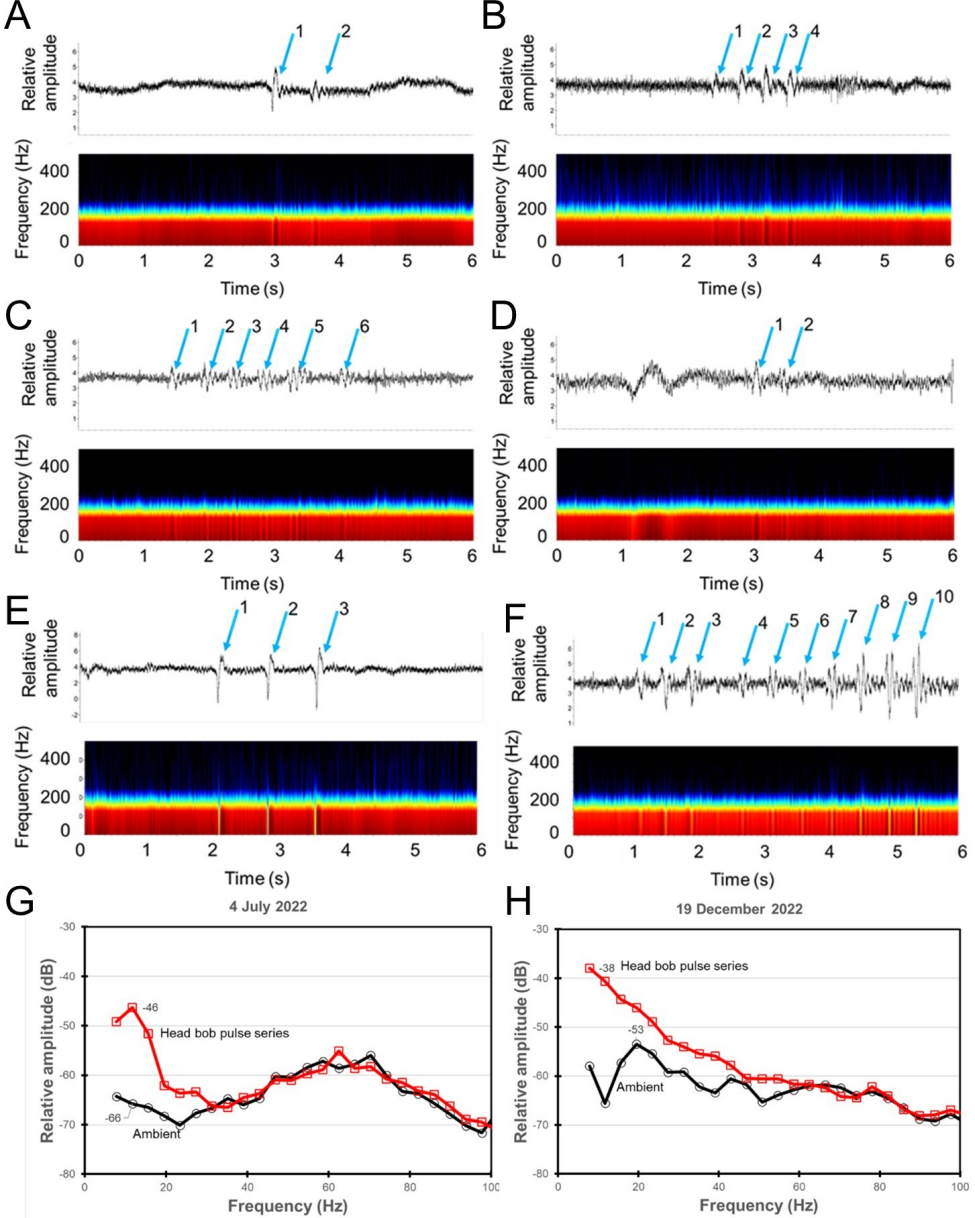
Six examples of the clearest low frequency pulses corresponding to head bobbing motions of seals in close pursuit of sablefish. Each panel contains the waveform (relative dB) on top and spectrogram on the bottom (1024 point Hann windowed FFT with 50% overlap, 2 kHz filter). Time is in UTC. A) June 28, 2022 at 17:11 (UTC) 2 head bobs, sablefish out of view but dust cloud suggests startle or strike; B) July 4, 2022 at 02:11 (UTC) four pulses, last two correspond to 2 head bobs as seal enters view chasing sablefish, sablefish startles; C) July 4, 2022 at 12:14 (UTC) six pulses corresponding to six head bobs as seal chases sablefish, last head bob occurs as seal move off screen, sablefish out of view but evasive; D) July 24, 2022 07:12 (UTC) two pulses corresponding to 2 head bobs as seal chases sablefish, sablefish strong startle. E) December 19, 2022 at 04:14 (UTC) 3 pulses corresponding to 3 head bobs as seal chases sablefish close to hydrophone, sablefish offscreen but evasive; F) May 15, 2023 at 05:14 (UTC) 10 pulses corresponding to 10 head bobs as seal approaches sablefish which does not startle but glides slowly (no fin movements) to shelter under instrument platform. G) and H) Comparison of the normalized relative power spectra of sounds produced during head bobbing (Hanning FFT 32768 samples, spectral resolution 3.9 Hz) on two dates: G) July 4, 2022 at 2:14 (UTC, spectrogram shown in C), H) December 19, 2022 at 04:14 (UTC, spectrogram shown in E).

The local soundscape at the FAAE site was unexpectedly dominated by instrument noise primarily from the sonar which generated a broadband ping approximately every second when not recording (standby mode), and 4 pings/s when recording (Fig 7A). Each ping contained eight pulses, approximately 103 ms in duration, and from approximately 1600 Hz to 58000 Hz in standby mode, and 55 ms duration and 2100–58000 kHz when scanning. Pings contained harmonics under both conditions, with significant elevation above the ambient at 4 kHz and when recording, the noise produced by the sonar fell in the hearing range of the elephant seal (Fig 7B). Interestingly, underwater sound detection thresholds have only been obtained for one juvenile female northern elephant seal [60]; assuming possible differences in auditory sensitivity between individuals and/or across sex, we cannot exclude that the sonar was also within hearing range of the elephant seals when in standby. The bait release system also produced detectable sounds as the carousel turned to release bait, but only once every 14 days (not shown). Sounds were not available for 3 of the 113 videos. The soundscape was remarkably quiet, except for the frequent occurrence of distant ship noise and cetacean calls. Most interestingly, the occurrence of low frequency pulses corresponding to seal head bobbing behavior were observed in ten of the eleven times when head bobbing behavior was observed in the videos (S2 Table, Fig 8A–8F, S7, S10–S12, S14, S15 Videos). However, sounds were not detected during one head bobbing observation (S13 Video). In one case when a seal was bobbing its head as it entered the FOV, four pulses were observed for two head bobs suggesting the first two occurred offscreen (S11 Video and S2 Table). In the clearest example, the elephant seal makes ten head bobbing motions while in close pursuit of a sablefish (S15 Video) which corresponded to ten low frequency pulses (Fig 8G). On 19 December 2022 five separate head bobbing events were observed from the same seal, but four had poor sound quality. The last event where the seal was in close proximity to the hydrophone produced three strong pulses corresponding to the three head bobs (Fig 8E and S14 Video). Although the peak frequency is obscured in the background noise, a comparison of relative power spectra between the pulses and ambient, suggests sounds below 40 Hz with a peak around 12 Hz about 20 dB above the ambient (Fig 8G and 8H).

Sounds were also useful in elucidating behavior observed in the video (S6 Fig and S1–S6 Videos). Struggle sounds associated with sablefish capture (August 3, 2022 at 06:14 UTC, S1 Video) were similar to sounds associated with a suspected capture just off-screen on October 29, 2022 at 18:16 UTC (S3 Video). Sounds of the elephant seal snatching up a suspected squid prey were also detected (S6 Fig and S2 Video). No sounds from targeted (including sablefish) or potential prey were detected.

## Discussion

Our findings provide novel insight into sub-adult male northern elephant seal presence, their prey selection and foraging strategies, as well as their behavior in a deep-sea (645 m depth) benthic environment of the Northwestern pacific (i.e., Barkley Canyon node). We show that from late June 2022 to mid-May 2023, at least eight (six recurrent and two temporary) sub-adult male northern elephant seals visited the same cabled observatory location (the FAAE platform), spending some time on the sea bottom, resting and foraging primarily on sablefish. The enforced multiparametric monitoring strategy [ie., combining video, sonar, and passive acoustics into an ethologically oriented sensor package; 30, 31] provided information that together expands our insight into the elephant seal at-sea ethology more so than any instrument type alone. It is important to acknowledge that both HD camera and sonar recorded only a small proportion of the area around the site (HD: ~12m^2^; sonar: ~25m^2^) and one might expect elephant seals to frequently occur beyond the imaging reach of the cabled observatory platform, meaning that their presence at this site might have been underestimated in our study. However, to our knowledge, these videos are unique observations of deep-sea free-ranging elephant seals, showing interactions with other species of the pelagic and benthic deep-sea community, as required for a reliable view of trophic web structures.

### Elephant seal occurrence at Barkley Canyon deep-sea node

Northern elephant seals were found at the site during seven distinct periods between June 22, 2022 and May 19, 2023. Those periods lasted between 6 and 54 days and were between 10 to 108 days apart. Their visits did not correspond to the bait release dates (except on one occasion), which might have attracted more potential prey, and were not always aligned with lower maximum daily tidal range and/or new moon phase. This potentially means that there are other seasonal drivers for the presence periods. Biotic factors, such as elephant seal migration, individual or age variation, seasonal differences in prey availability (see Fig 5C), competition with other top predators or predation risks, could also influence the patterns we observed. Interestingly, Naito et al. [11] reported that female northern elephant seals foraged in the deep oxygen minimum zone, where prey mobility may be reduced due to the low oxygen concentrations. Nevertheless, at the FAAE site, although the oxygen concentration was also low (approximately 0.1–0.6 ml/l; corrected), sablefish at this location exhibited an escape response from the elephant seals, suggesting that the observed elephant seals did not benefit from reduced prey mobility. Sablefish are known to have high tolerance to low dissolved oxygen and occur in low dissolved oxygen areas, but dissolved oxygen still has an effect [61, 62]. Interestingly, the period with the abrupt reduction in dissolved oxygen (P4) corresponded to a reduced sablefish presence, reduced foraging activity but increased resting activity for elephant seals (Mike), and the departure of those elephant seals happened concurrently with the lowest level of dissolved oxygen. Elephant seals (Mike) came back during P5 when both dissolved oxygen levels and sablefish presence increased, and they resumed their foraging activity.

Eight sub-adult male elephant seals were identified in the HD videos. At least six of those individuals made repeated visits (> 2) to the same site over multiple days suggesting that the Barkley Canyon deep-sea node was used as a Focal Foraging Area [FFA; 12]. FFAs are locations where individuals stop travelling and linger for long periods, continuing to dive and apparently foraging intensively. Adult males travel to coastal areas and forage on the continental shelf, including off of Washington state and British Columbia [12, 24]. However, the presence of only sub-adult males near Barkley Canyon Node, a site closer to their rookeries (from central Baja California to Oregon), might be explained by the foraging trip timing of sub-adult vs adult males and/or a higher probability of encountering sub-adult males given the higher mortality rates of adult males [24, 63]. The absence of sub-adult male elephant seals from February to early May at the FAAE site overlaps the end of the breeding season (Feb and early March) and only partly overlaps the molting season for sub-adult males (May). Juvenile males (those who have not yet developed secondary sexual characteristics) start molting in late March and through April and May, but the older/larger males generally molt later into the spring. Interestingly, in our study, the presence of sub-adult male northern elephant seals along the coast of Vancouver Island overlap with key migration routes of a mix between Alaskan and West coast sablefish stocks during sablefish migrations [e.g., 64, 65]. Future studies examining correlations between sablefish and elephant seal migrations might provide insight into elephant seal migration patterns and foraging behavior.

The unexpected and repeated occurrence of elephant seals at the FAAE site suggests that they were attracted to and able to precisely locate a small site (< 100 m^2^ area) in the open ocean in complete darkness on multiple occasions. An innate geospatial orientation is a possible explanation, but it would require the ability to maintain a precise location while at the surface despite a lack of a spatial reference frame and strong ocean currents. However, male northern elephant seals are known to travel to coastal habitat along the eastern North Pacific coastline and feed continuously in localized foraging areas [mean size of 7892 ± 4369 km2, which is about 88 km on a side; 12] over the continental shelf, suggesting some spatial fidelity. A second explanation is that by simply following large sablefish aggregations at Barkley Canyon Node, elephant seals remain in a general area and are led to the FAAE site by the sablefish returning from their diel vertical migrations [66]. Elephant seals are also known to exhibit Area-Restricted Search (ARS) behavior to optimize foraging [67]. A third explanation is that the seals homed in on the constant pulsed broadband noise (with harmonic structure) generated in 2022 by the sonar itself (Fig 7). Based on northern elephant seal audiograms [Fig 7, 60] and the sensitivity of seals and sea lions to harmonic signals [68], northern elephant seals in our study likely could have detected the sonar noise, which might explain how elephant seals were able to locate precisely the FAAE site for long periods (11–30 days). Interestingly, after sonar failure elephant seals were still observed on repeated occurrences but only for short periods (3–4 days), suggesting that elephant seals might use another cue to still find the FAAE site for short periods. Deep-diving elephant seals have blue-shifted rod opsin pigments, which provide increased sensitivity to the blue-green wavelength [λ ∼487 nm [69]], facilitate detecting and foraging bioluminescent prey (e.g., myctophid fish) [27] and might allow them to detect the light field produced by the LED. When the sonar was functional, most of the elephant seals were already present on site, as observed on sonar imagery and as observed with the timing of the first appearance on HD video (median = 1.3 s). In contrast, elephant seals were observed later in the HD camera recordings (median = 154 s) for periods when the sonar had failed, suggesting that elephant seals were still in the area and might be guided to the FAAE site by LED lights turned on.

Many fish are attracted to structures, and it has been suggested that ocean observatories act as artificial reefs [30]. If elephant seals in our study were indeed attracted to the active pings emitted by the sonar, our findings suggest that the elephant seals learn to associate the specific “soundscape” location with food availability. Similarly, other seal species have been suggested to be able to learn to use anthropogenic noise as an indicator of food location, also known as the “dinner bell” effect [70]. For example, [71] and [72] demonstrated that grey seals (*Halichoerus grypus*), harbor seal and sea lions learned to use information provided by acoustic tags to locate tagged fish.

### Elephant seal sensory adaptation and possible exaptation

The deep-diving northern elephant seal visual system, used while diving to locate and capture prey, is designed to function in dimmer conditions and to respond to greater changes in light levels than shallower diving pinnipeds [73]. Their higher light sensitivity [74] might explain the strong behavioral reaction from elephant seals when FAAE LED lights were turned on just before the HD recordings; with many of the resting elephant seals leaving the area immediately or almost immediately. However, it seems that, after a short adaptation period, the lights did not affect their foraging ability with many of the elephant seals observed actively pursuing prey in the videos (49%). Lights are known to have a strong effect on fish behavior, and are thought to alternatively attract and repel sablefish in the short term [<60s; 55]. Frequent startle responses observed at the site in 2019 [75, 76] also suggest that lights affect sablefish behavior. Therefore, one interpretation is that elephant seals are attracted to the FAAE site due to the availability of prey and use the FAAE as a foraging and resting site, but then take advantage of fish disturbance caused by the artificial lights to improve foraging success. Under this scenario elephant seals might engage in both resting and foraging behavior (searching, pursuing and capturing prey) at any time, but increase prey pursuit, and potentially foraging success, when the lights disturb the sablefish.

Sensory abilities that supplement underwater vision have evolved in deep-diving predators. Pinnipeds are whisker specialists, and whisker control (movement and positioning) is an important aspect of touch sensing in these animals [77]. Recently, Adachi et al. [27] demonstrated that elephant seals rely primarily on hydrodynamic prey sensing using their whiskers to aid in searching, pursuing, and capturing prey. In our study, elephant seals oriented their head towards the moving fish target and protracted their whiskers when prey were present, confirming that elephant seals actively use their vibrissae system for prey sensing [27]. Some species, such as rockfish observed in our videos, adopt predator avoidance behavior by staying immobile most of the time. Our observations suggest that seals appear to ignore stationary or drifting prey but primarily focus on actively swimming prey, which create hydrodynamic trails likely to be detected by elephant seals through their vibrissae [78]. Naito et al. [11] suggested that larger elephant seals tended to feed on larger prey to satisfy their metabolic needs. Although in our observations most drifting prey (e.g., rattails and eelpouts) were small individuals less likely to be of interest to the seal, it is also possible that they were just undetectable as drifting prey. On at least two occasions, a seal switched its attention from a sablefish it was slowly chasing to another nearby fish that had accelerated in a startle response (one a sablefish, the other a snailfish; see S4 and S7 Videos). In another case, a seal reacted strongly to a sablefish that had been resting beneath the bait dispenser carousel after it startled and darted away (see S8 Video).

Our observation of apparent (voluntary or involuntary) infrasonic sound production by the elephant seals in pursuit of sablefish prey suggests its use in an underwater foraging context. In seven of the eleven instances of head bobbing behavior, sablefish were observed to startle strongly at the same time (see S2 Table, S7, S11, and S13, S14 Videos) or slightly later (see S15 Video). In the other four instances the sablefish were offscreen, but elephant seal action and sediment stirred up from the sea floor also suggest a startle response. Although these startle responses may simply be a reaction to the closely approaching elephant seal, the sudden change in flight behavior corresponding to the elephant seal’s head bobbing suggest a possible response to the infrasound (see especially slow motions in S7 and S11 Videos). Fish are well known to be highly sensitive to infrasonic sound [e.g., 79–81] which can be used to induce startle responses or construct barriers [82, 83]. Elephant seals may produce an infrasonic sound to disturb the sablefish and potentially create a startle response to herd the prey into exhaustion, and/or to create a hydrodynamic trail. It is not known if the northern elephant seal can hear infrasonic sounds underwater but they possess a large and distinct nucleus ellipticus, similar to those found in the African elephant and cetaceans, which is thought to be important in infrasonic hearing [84]. The production of infrasonic sounds might be an example of an exaptation [85], where another process (e.g., head bobbing) leads to the development of sound production mechanisms [86]. Although our observations strongly suggest infrasonic sound production by northern elephant seals pursuing prey, future research is needed to confirm the behavior.

### Elephant seal foraging behavior and prey selection

Elephant seals are opportunistic generalist feeders with a broad foraging niche including cephalopods, teleosts, crustaceans, elasmobranchs, cyclostomes and tunicates [87, 88]. The availability of high-frequency (i.e., hourly) and long-term (1 year) video data from the seafloor NEPTUNE observatory provides a unique perspective on seal hunting strategies not available from animal borne cameras. Our observations of elephant seals following some distance behind a sablefish (which often had passed through the FOV by the time the seal entered), is consistent with reports that pinnipeds, such as harbor seals, can follow hydrodynamic trails as far away as 40 m in complete darkness [9, 78, 89]. This strategy might serve to slowly tire out the sablefish without the elephant seal having to expend a lot of energy in the chase. Another advantage of benthic foraging is that males could trap prey against the sea bottom (as observed on videos), possibly improving prey capture rate and reducing pursuit effort. On two occasions the seal appeared to take advantage of the sablefish being hindered by the observatory cable (see S1 and S3 Videos), while in other cases the sablefish used the structure to evade the seal (e.g., S16 Video).

Sablefish are known to be one of the most abundant fishes attracted to other locations within the Barkley Canyon Observatory system [37, 55, 90–92]. Our video observations demonstrated that most sub-adult northern elephant seals foraged predominantly on sablefish on the bottom, suggesting that this species is an important food item. Sablefish would likely be a high-quality prey for elephant seals, being large and rich in lipids [93], with the skull containing large quantities of oil (60% lipid dry weight) and triglycerides being the primary lipid component (97% of the lipid). Interestingly, in our study, elephant seals appeared to actively avoid a snailfish and even rejected one already caught (see S7 Video). This is an interesting reaction for an animal known to sometimes even consume hagfish (despite their intensive slime production) and elasmobranchs [87], and suggests that snailfish may possess some type of antipredator physiology.

Kienle et al. [24] reported a bottom time ~ 12 min for males during their benthic dives, a value similar to our maximum bottom time observed (876 s or 14 min 36 s). Although reported male northern elephant seal maximum depth is 1,529 m [94], Le Boeuf et al. [12] demonstrated that overall adult males dove most often to depths of 312 ± 117 m, which is less deep than typical adult female dives (456 ± 52 m). Thus, the repeated presence of sub-adult male northern elephant seals at 645 m deep is an interesting observation, suggesting that the FAAE site might be a driver for deeper than usual dives. The slightly higher (but not statistically different) occurrence of sub-adult male elephant seals at the observatory during daytime compared to nighttime matches the distinctive diel vertical movements of mesopelagic prey (including sablefish [55, 66]) and has been previously reported in northern elephant seals [24, 28, 94].

### Elephant seal resting behavior

Male northern elephant seals perform more flat-bottomed (benthic) dives than females, which perform pelagic dives more often [25, 95]. Our observations on multiple occasions of sub-adult male northern elephant seals staying immobile at the bottom are in agreement with previous observations of adult males ceasing swimming in a subset of flat-bottomed dives [95] and suggest that male northern elephant seals also slept at depth. Le Boeuf et al. [16] suggested that sleeping near the bottom of dives would provide security when faced with near-surface (< 150 m deep) predators such as killer whales or great white sharks. Mitani et al. [21] reported that, in cases of drift-and-bottom-rest dives, after reaching the seafloor, elephant seals laid immobile for 4.8 ± 3.1 min (0.6–8.0 min) with no reaction to the sudden shock upon contact with the seafloor. Those values are similar to the “no motion” bottom time observed in our study and to our maximum “resting” duration (8 min 40 sec). In our study, northern elephant seals showed inactivity (no flipper strokes), a stereotypical posture when laying down and a reduced responsiveness to external stimulation (when lights were not turning on directly in the elephant seal’s face, S18 video), suggesting that elephant seals were actually sleeping or resting [96], but this hypothesis cannot be confirmed without using more traditional methods (e.g., recording the electroencephalogram or eye closure).

## Conclusions

Our findings based on multi-sensor observations at the FAAE experimental site in Barkley Canyon deep-sea node demonstrate the utility of designing marine observatories with spatially and temporally cross-referenced data collection from instruments representing multiple modalities of observation [30, 31]. The data collected by the ONC cabled video-observatory at the Barkley Canyon Node provided evidence that sub-adult male northern elephant seals are present in British Columbia from June to January, are absent from February to April and return in May. At least six sub-adult male northern elephant seals visited the same cabled observatory location (the FAAE platform) at 645 m deep over multiple days (> 2 days). Four of them were able to locate precisely the FAAE site for long periods (11–30 days), possibly due to the noise generated by the sonar (which was revealed by the continuous recording of the local soundscape), suggesting that they learned to use anthropogenic noise as an indicator of food location, also known as the “dinner bell” effect. The HD camera provided novel and highly valuable information regarding foraging behavior and prey selection of sub-adult male northern elephant seals. Multi-beam imaging sonar can function as a complementary tool to cabled observatory systems in dark and deep waters providing underwater acoustics images of targets. Recordings by both the sonar and conventional HD camera at the site showed that 1) the elephant seals were attracted to the site and appeared to take advantage of the light attraction and startling effects on sablefish to enhance foraging success; and 2) they were sleeping or resting at depth. During foraging activity elephant seals primarily focused on actively swimming sablefish, ignoring stationary or drifting potential prey, and produced (voluntary or involuntary) infrasonic sounds when pursuing prey (as revealed by the incorporation of audio with the underwater video). Because we only report opportunistic observations (“snapshots”) of sub-adult male northern elephant seal presence and behavior at one site (the observatory at Barkley Canyon Node), our ability to extrapolate these results to the entire sub-adult male northern elephant seal population is limited. This study highlights the utility of designing marine observatories with spatially and temporally cross-referenced data collection from instruments representing multiple modalities of observation. Further work is currently being carried out by ONC in the development of analytical tools that better integrate the recordings and processing of independent data streams such as video, passive acoustics, sonar, and other sensor data, including camera embedded machine learning pipelines [e.g., 97, 98] that will greatly enhance research on deep-sea animal behavioral ecology [99].

## Supporting information

S1 Fig Configuration of the FAAE experiment and sampling protocol. A) Side view of the FAAE platform. B) Video camera field of views when completely zoomed out (~12 m^2^, B1), and when zoomed in and focused on the bait release carousel (~6 m^2^, B2). C) Schematic illustrating the sampling routine and intervals of HD video, DIDSON sonar and IcListen HF hydrophone. D) Screenshot of fly through video captured by remotely operated vehicle Hercules showing the overall configuration of the experiment in the seafloor of Barkley Node, at 645 m depth.
(TIF)

S2 Fig HD video duration, DIDSON sonar maximum range, and elephant seal occurrence. A) HD Video duration (in minutes) and B) corresponding elephant seal events in each hour-bin (local time, UTC-8h, dark gray) (light grey illustrates data collection). C) DIDSON sonar maximum range (in meters) in each 15-min video and D) corresponding elephant seal events (in each hour-bin, dark gray) (light grey illustrates data collection). Failure of the DIDSON sonar is indicated by the absence of data collection after 11 November 2022 at 1500 LST (C and D). Seven periods (P1 to P7) are identified with elephant seal sightings.
(TIF)

S3 Fig Most common potential elephant seal prey identified in the HD videos. Most common potential elephant seal prey identified in the HD videos included: sablefish, hagfish, Dover sole, squid, brown catshark, rattail, giant Pacific octopus, crabs, unidentified fish, eelpout, rockfish, skate, snailfish and poacher. The species presented here are for illustration only and are representative of larger taxa groups.
(TIF)

S4 Fig Identification clues for each recognizable elephant seal individual. (TIF)

S5 Fig Environmental parameters, bait releases and elephant seal occurrence. A) Tide amplitude (m) and B) Maximum daily tidal range (m) at Tatoosh Island (Cape Flattery, WA, USA; NOAA Tide and Currents) near Barkley Canyon. At the top, circles indicate moon phases, and at the bottom, black dots indicate days with elephant seal presence. C) Oxygen concentration corrected (in ml/l, average; black line) at Barkley Node during the FAAE experiment. Grey lines represent daily minimum and maximum values, black dots indicate days with elephant seal presence. D) Diel and hourly occurrence of elephant seals at Barkley Canyon Node in 1-h bins (black boxes). Cyan areas illustrate bait release dates and time (every 14 days at 11 am, local time), and purple area illustrates bait release with concurrent elephant seal presence.
(TIF)

S6 Fig Examples of sounds produced as a seal attacks prey. Each panel contains the waveform (relative dB) on top and spectrogram on the bottom (1024 point Hann windowed FFT with 50% overlap). Note different time and frequency scales (All times are in UTC). A) 27 July 2022, 17:12 (UTC) seal bites at sablefish, 6 kHz filter (see S4 Video); B) 3 August 2022, 06:14 (UTC) seal catches sablefish, 2 kHz filter (see S1 Video); C) 6 August 2022, 01:13 (UTC) seal catches possible squid, filtered between 0.3 and 4 kHz (see S2 Video; D) 29 October 2022, 18:16 (UTC) seal possibly catches sablefish just offscreen, 2 kHz filter (see S3 Video).
(TIF)

S1 Table Classification based on Casey et al. (2020) of development stages for male northern elephant seals. (DOCX)

S2 Table The occurrence of head bob observed in the videos and corresponding low frequency pulses observed in the corresponding sound recording. Sablefish startle responses apparently evoked by the head bobbing behavior are indicated when the fish was in view.
(DOCX)

S1 Video August 3, 2022 at 06:14 (UTC). Seal catches sablefish against hydrophone cable (see S6B Fig).
(MP4)

S2 Video August 6, 2022 at 01:13 (UTC). Seal appears to catch a squid supported by sounds (see S6C Fig).
(MP4)

S3 Video October 29, 2022 at 18:16 (UTC). Possible sablefish catch by seal just offscreen, supported by struggle sounds (see S6D Fig).
(MP4)

S4 Video July 27, 2022 at 17:10 (UTC). Seal flanks sablefish, but ignores a resting sablefish as it passes. It then attacks a 3nd sablefish moving in from offscreen. Sounds suggest capture attempt or capture (S6A Fig).
(MP4)

S5 Video May 16, 2023 at 04:16 (UTC). Seal sneaks on a sablefish and traps it.
(MP4)

S6 Video May 17, 2023 at 18:18 (UTC). Seal catches sablefish.
(MP4)

S7 Video July 24, 2022 at 07:12 (UTC), seal rejects snailfish, then later makes 2 head bobs and 2 pulses while chasing sablefish, then rejects captured snailfish. Sablefish strongly startled by head bobs.
(MP4)

S8 Video 24 July 2022 at 07:14 (UTC), seal approaches bait carousel and startles sablefish resting beneath, then pursues. (MP4)

S9 Video Three examples of seal apparent reactions to lights A) seal on bottom immediately leaves area and not seen again on video, B) delayed reaction of resting seal which wakes up and leaves after about 7 s, C) resting seal wakes up and leaves after 3 min 28 s (on the Axis video).
(MP4)

S10 Video June 28, 2022 at 17:11 (UTC), seal makes 2 head bobs while chasing sablefish offscreen. (MP4)

S11 Video July 4, 2022 at 02:11 (UTC), seal entering view chasing sablefish makes 2 head bobs corresponding to last 2 of 4 pulses. Sablefish is strongly startled.
(MP4)

S12 Video July 4, 2022 at 12:14 (UTC), seal makes 6 head bobs corresponding to 6 pulses while chasing sablefish. Sablefish is offscreen, but seal movements and dust cloud suggest a strong startle response.
(MP4)

S13 Video October 10, 2022 at 02:15 (UTC), seal makes 3 head bobs while chasing sablefish. Sablefish startles. Infrasonic pulses not detected.
(MP4)

S14 Video December 19, 2022 at 4:14 (UTC), seal makes 3 head bobs while chasing sablefish. Sablefish startles.
(MP4)

S15 Video May 15, 2023 at 05:14 (UTC), seal makes 10 head bobs while chasing sablefish. Sablefish has a delayed startle response.
(MP4)

S16 Video 5 September 2022 at 01:13 (UTC), Elephant seal snatches at and misses sablefish, supported by sounds. (MP4)

S17 Video 5 September 2022 at 21:13 (UTC), elephant seal snatches at sablefish just offscreen, supported by sounds. (MP4)

S18 Video Resting seal recorded on both DIDSON and HD videos (same event that the third example in S9 Video). (MP4)
