# Supplementary figures and images for: Deep-sea cabled video-observatory provides insights into the behavior at depth of sub-adult male northern elephant seals, Mirounga angustirostris
Source: PLoS One. 2024 Sep 4;19(9):e0308461. doi: 10.1371/journal.pone.0308461 (PMC11373836; doi:10.1371/journal.pone.0308461)

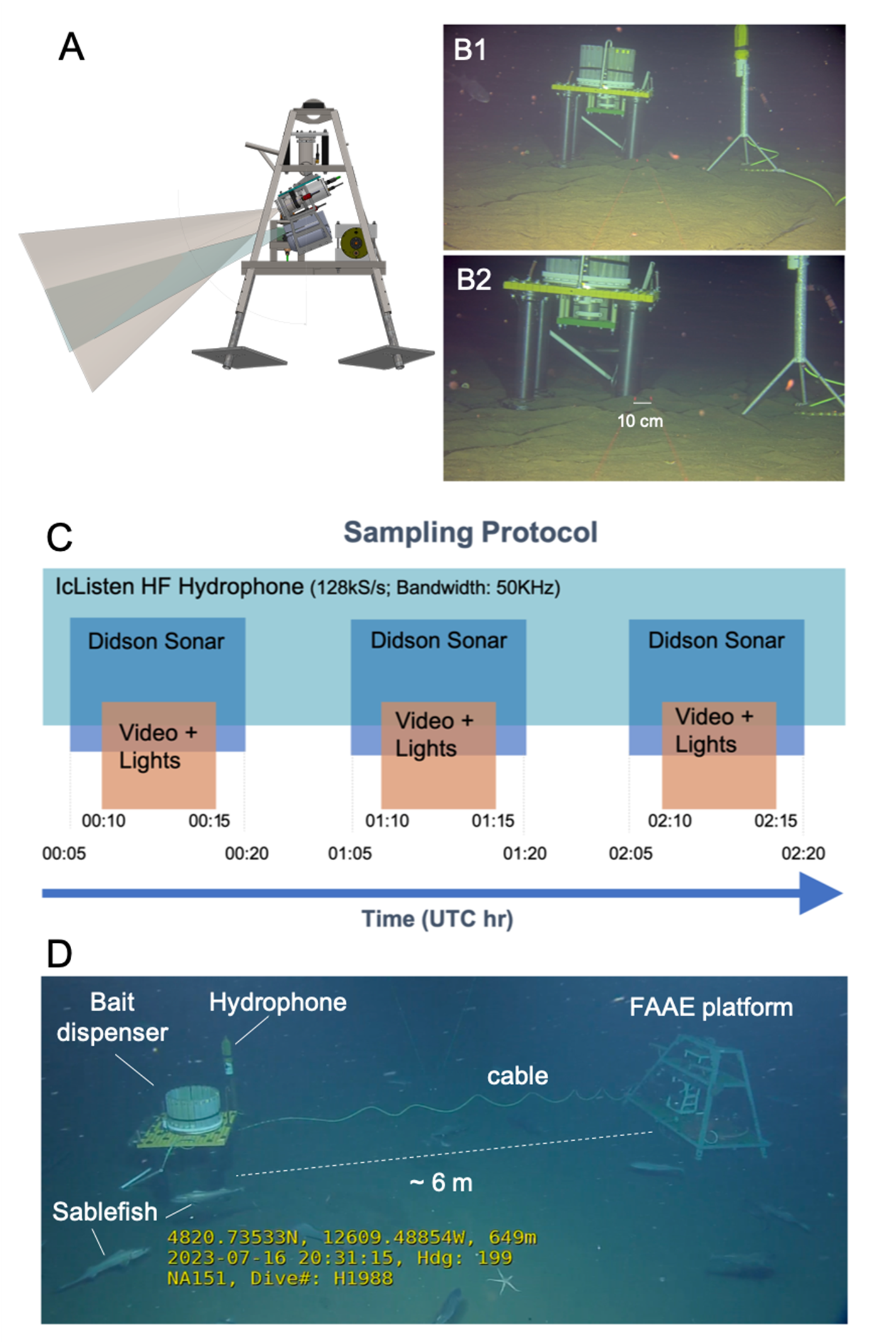

Supplement: S1 Fig — A) Side view of the FAAE platform. B) Video camera field of views when completely zoomed out (~12 m2, B1), and when zoomed in and focused on the bait release carousel (~6 m2, B2). C) Schematic illustrating the sampling routine and intervals of HD video, DIDSON sonar and IcListen HF hydrophone. D) Screenshot of fly through video captured by remotely operated vehicle Hercules showing the overall configuration of the experiment in the seafloor of Barkley Node, at 645 m depth. (TIF) [file pone.0308461.s001.tif]

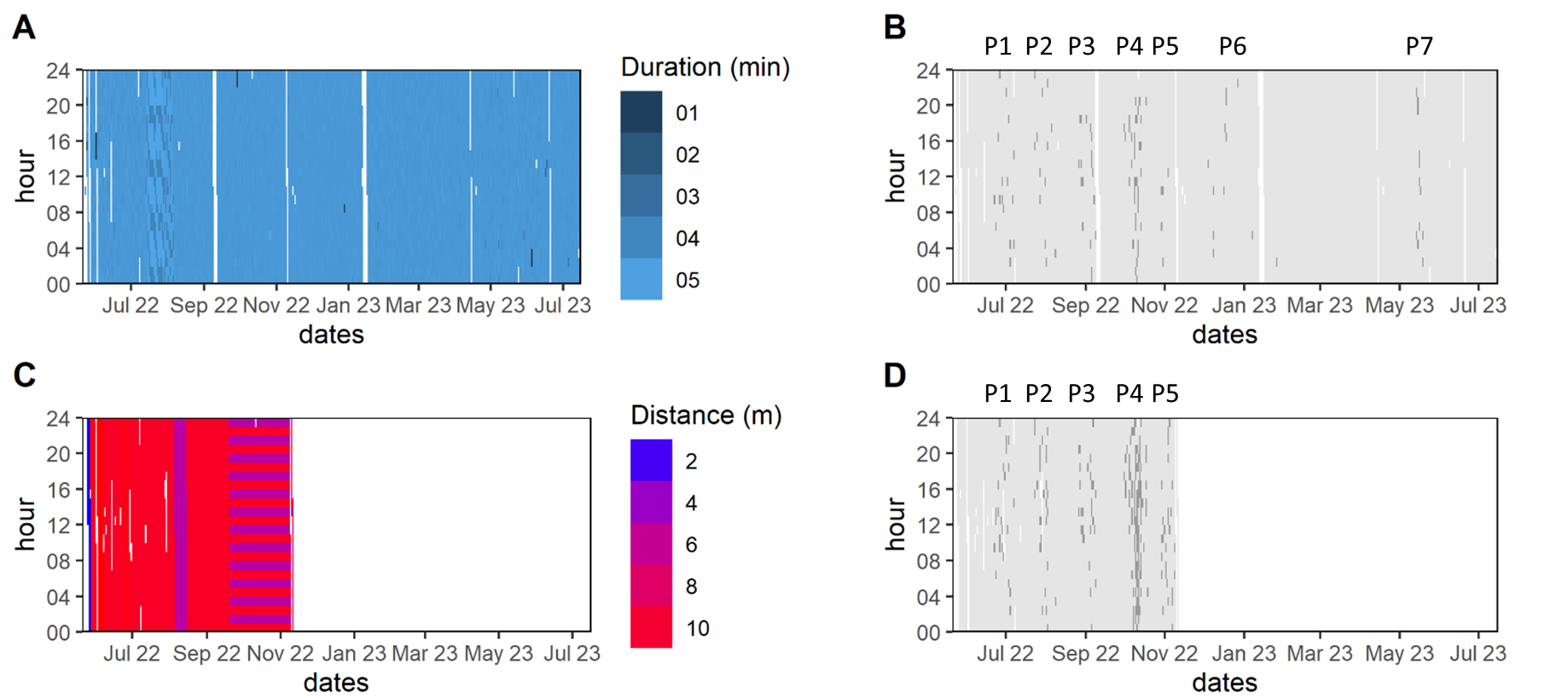

Supplement: S2 Fig — A) HD Video duration (in minutes) and B) corresponding elephant seal events in each hour-bin (local time, UTC-8h, dark gray) (light grey illustrates data collection). C) DIDSON sonar maximum range (in meters) in each 15-min video and D) corresponding elephant seal events (in each hour-bin, dark gray) (light grey illustrates data collection). Failure of the DIDSON sonar is indicated by the absence of data collection after 11 November 2022 at 1500 LST (C and D). Seven periods (P1 to P7) are identified with elephant seal sightings. (TIF) [file pone.0308461.s002.tif]

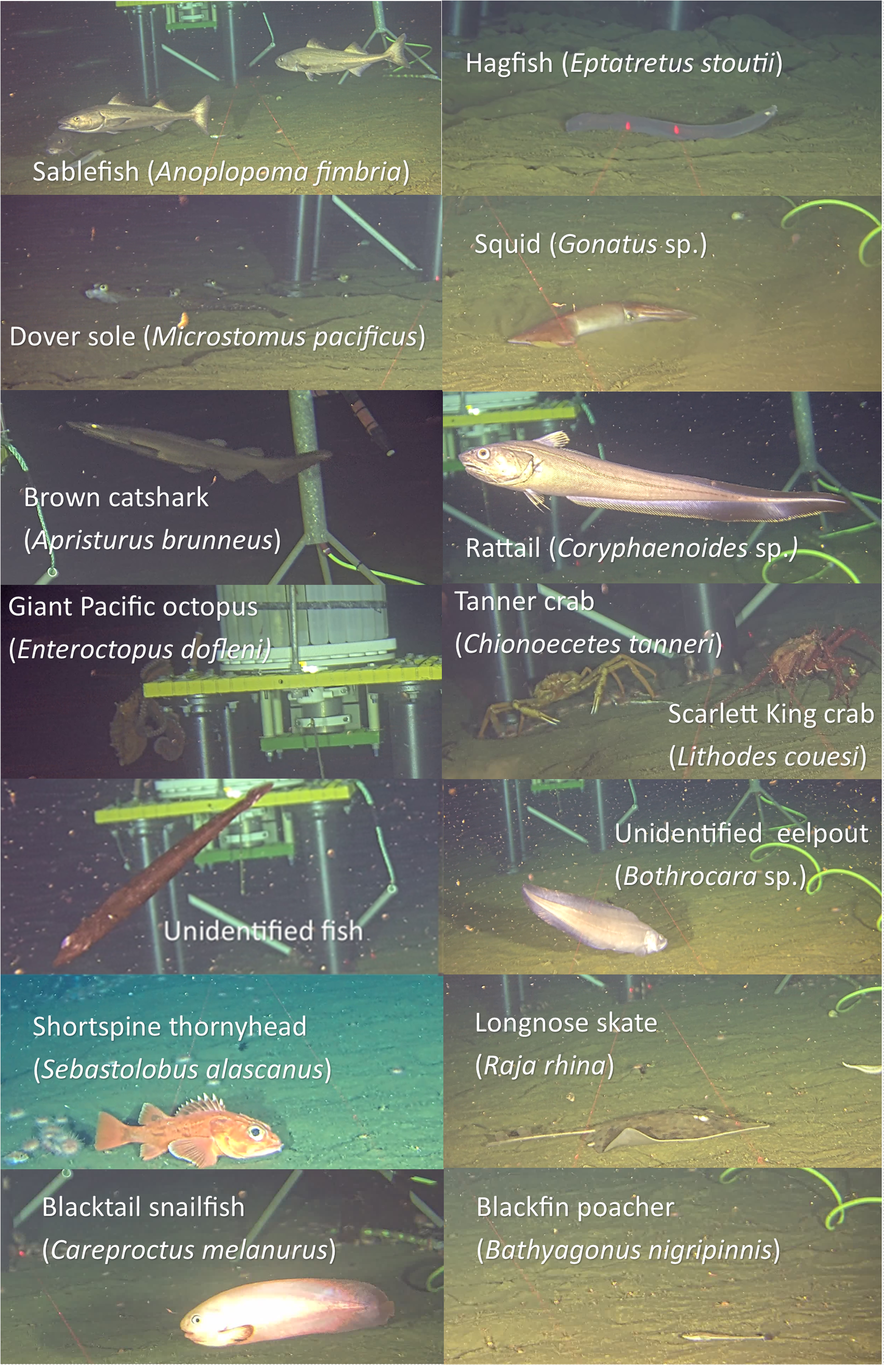

Supplement: S3 Fig — Most common potential elephant seal prey identified in the HD videos included: sablefish, hagfish, Dover sole, squid, brown catshark, rattail, giant Pacific octopus, crabs, unidentified fish, eelpout, rockfish, skate, snailfish and poacher. The species presented here are for illustration only and are representative of larger taxa groups. (TIF) [file pone.0308461.s003.tif]

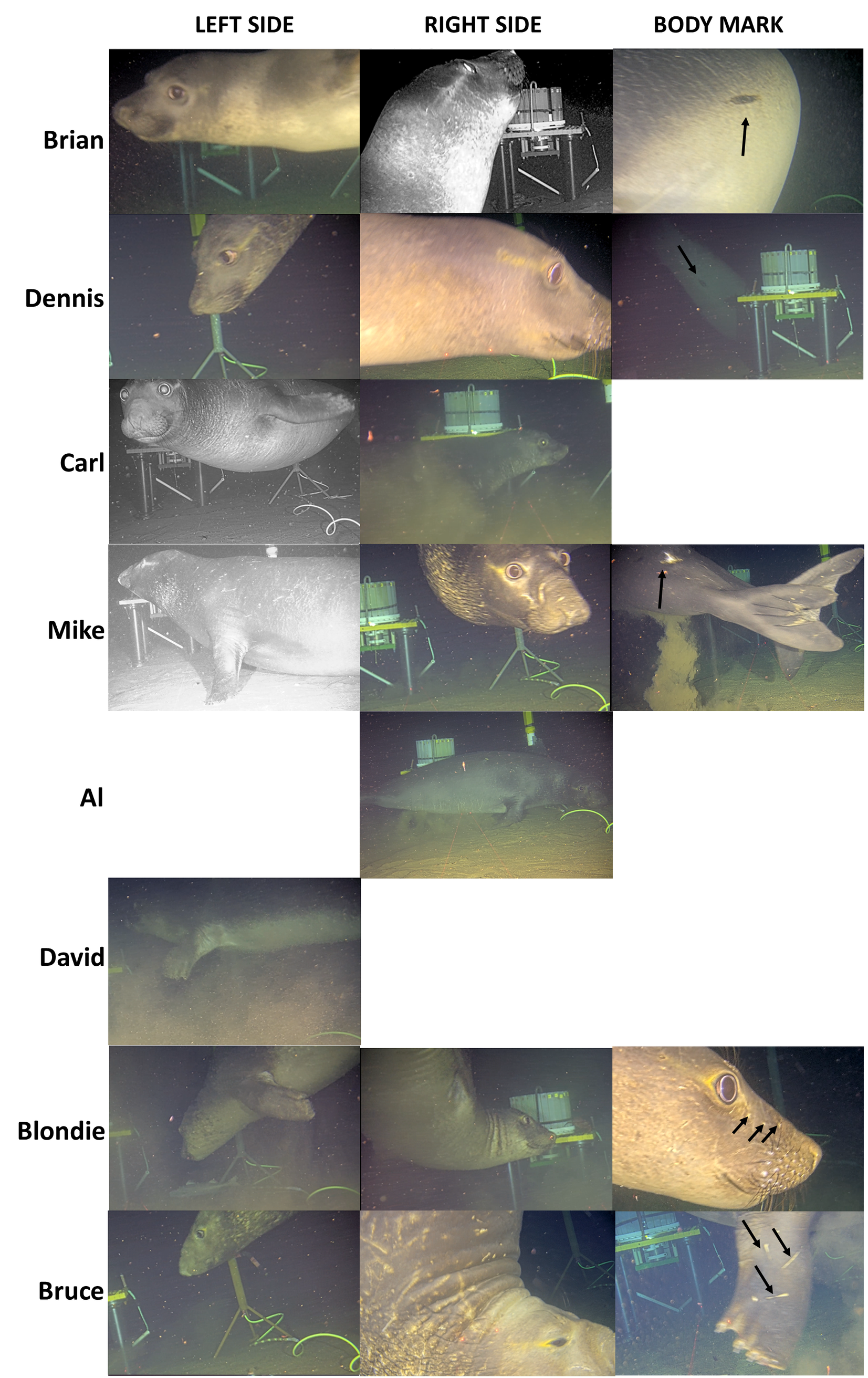

Supplement: S4 Fig — (TIF) [file pone.0308461.s004.tif]

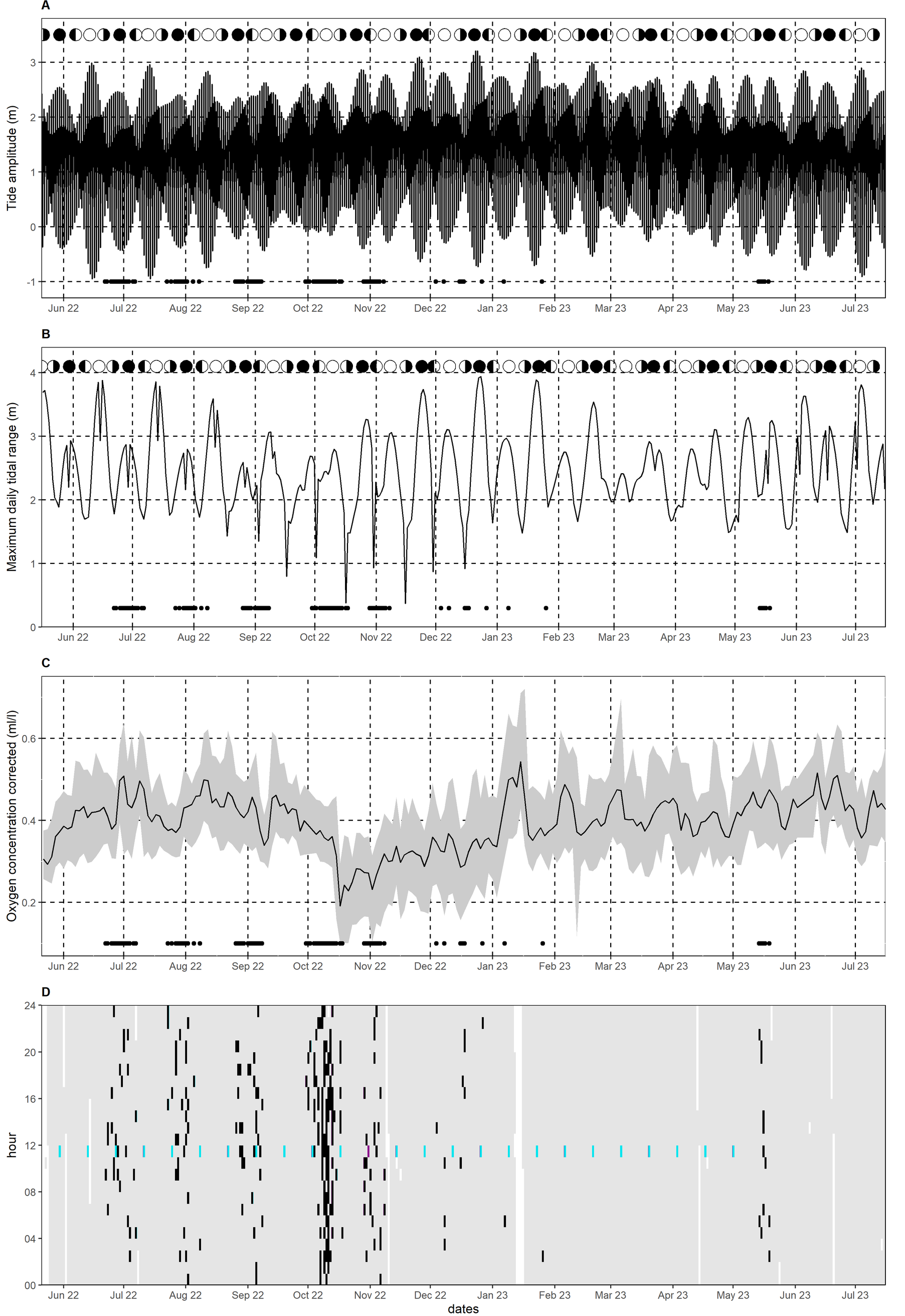

Supplement: S5 Fig — A) Tide amplitude (m) and B) Maximum daily tidal range (m) at Tatoosh Island (Cape Flattery, WA, USA; NOAA Tide and Currents) near Barkley Canyon. At the top, circles indicate moon phases, and at the bottom, black dots indicate days with elephant seal presence. C) Oxygen concentration corrected (in ml/l, average; black line) at Barkley Node during the FAAE experiment. Grey lines represent daily minimum and maximum values, black dots indicate days with elephant seal presence. D) Diel and hourly occurrence of elephant seals at Barkley Canyon Node in 1-h bins (black boxes). Cyan areas illustrate bait release dates and time (every 14 days at 11 am, local time), and purple area illustrates bait release with concurrent elephant seal presence. (TIF) [file pone.0308461.s005.tif]

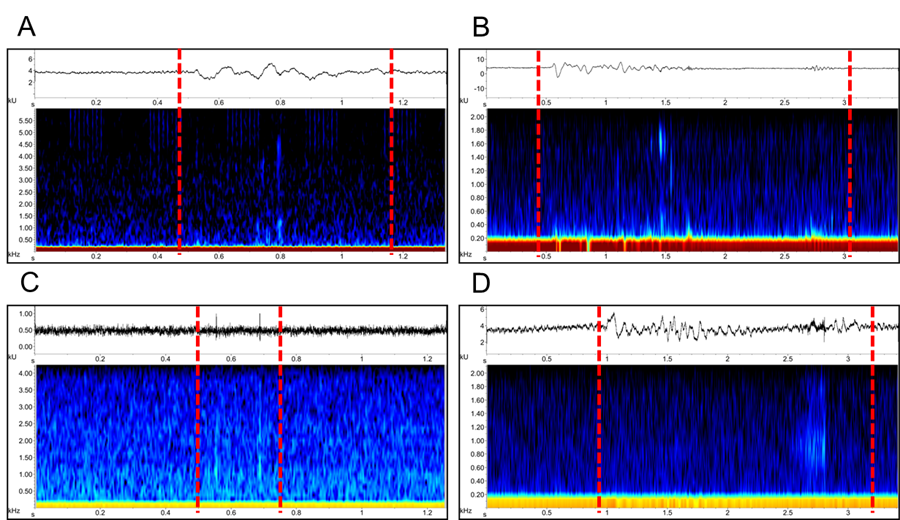

Supplement: S6 Fig — Each panel contains the waveform (relative dB) on top and spectrogram on the bottom (1024 point Hann windowed FFT with 50% overlap). Note different time and frequency scales (All times are in UTC). A) 27 July 2022, 17:12 (UTC) seal bites at sablefish, 6 kHz filter (see S4 Video); B) 3 August 2022, 06:14 (UTC) seal catches sablefish, 2 kHz filter (see S1 Video); C) 6 August 2022, 01:13 (UTC) seal catches possible squid, filtered between 0.3 and 4 kHz (see S2 Video; D) 29 October 2022, 18:16 (UTC) seal possibly catches sablefish just offscreen, 2 kHz filter (see S3 Video). (TIF) [file pone.0308461.s006.tif]
