# Supplementary material for: Deep-sea cabled video-observatory provides insights into the behavior at depth of sub-adult male northern elephant seals, Mirounga angustirostris
Source: PLoS One. 2024 Sep 4;19(9):e0308461. doi: 10.1371/journal.pone.0308461 (PMC11373836; doi:10.1371/journal.pone.0308461)
Supplement: S1 Table — (DOCX) [file pone.0308461.s007.docx]

| **Male age class** | **Age** | **Size / weight** | **Secondary sexual characteristics** |
| --- | --- | --- | --- |
| Sub-adult 1 (SA1) | 4 years old | Similar to adult females (up to 600 kg) | Nose: wide, not elongated, no mid-nose indentation  Chest: no scarring |
| Sub-adult 2 (SA2) | 5 years old |  | Nose: proboscis that extends down to the mouth when the seal rests flat on the ground, no mid-nose indentation  Chest: wrinkles along the chest shield, no scarring |
| Sub-adult 3 (SA3) | 6 years old |  | Nose: proboscis extends down past the mouth while resting on the ground, mild mid-nose indentation  Chest: Scarring of the chest shield, thickened skin and some wrinkles but no pink tissue |
| Sub-adult 4 (SA4) | 7 years old |  | Nose: proboscis that folds onto the ground while resting, significant mid-nose indentation  Chest: Scarring along the chest that has some pink coloration, chest shield can extend proximally towards the eye while observed resting on the ground |
| Adult | 8+ years old | Can weigh over 2000 kg | Node: wide proboscis with pronounced mid-nose indentation; while at rest, the nose extends to the ground and folds back under the mouth  Chest: Noticeable calloused pink chest shield that extends proximally past the eyes and up along the dorsal side of the head |
