# Supplementary material for: Deep-sea cabled video-observatory provides insights into the behavior at depth of sub-adult male northern elephant seals, Mirounga angustirostris
Source: PLoS One. 2024 Sep 4;19(9):e0308461. doi: 10.1371/journal.pone.0308461 (PMC11373836; doi:10.1371/journal.pone.0308461)
Supplement: S2 Table — Sablefish startle responses apparently evoked by the head bobbing behavior are indicated when the fish was in view. (DOCX) [file pone.0308461.s008.docx]

| Date (UTC) | Hour (UTC) | Video elasped time | Head bobs | Low frequency pulses | Startle response | Sound quality | Comment | Online video |
| --- | --- | --- | --- | --- | --- | --- | --- | --- |
| 6/28/2022 | 17 | 1:45 | 2 | 2 | offscreen | good | 2 pulses corresponding to 2 seal head bobs | S10 |
| 7/4/2022 | 2 | 0:46 | 2 | 4 | yes | good | 2 head bobs occur as seal enters view but 4 pulses suggest 2 head bobs occurred offscreen prior to entry | S11 |
| 7/4/2022 | 12 | 4:00 | 6 | 6 | offscreen | good | 6 pulses corresponding to 6 seal head bobs | S12 |
| 7/24/2022 | 7 | 2:19 | 2 | 2 | yes | good | 2 pulses corresponding to 2 seal head bobs | S7 |
| 10/10/2022 | 2 | 1:06 | 3 | 0 | yes | poor | no clear pulses detected | S13 |
| 12/19/2022 | 4 | 1:19 | 3 | 3 | delayed | weak | 3 pulses corresponding to 3 seal head bobs, seal in background | none |
| 12/19/2022 | 4 | 1:36 | 3 | 3 | yes | poor | signal distorted | none |
| 12/19/2022 | 4 | 1:54 | 2 | 1 | yes | poor | seal far from hydrophone, only 1 weak pulse detected | none |
| 12/19/2022 | 4 | 2:00 | 3 | 1 | offscreen | poor | seal far from hydrophone, only 1 weak pulse detected | none |
| 12/19/2022 | 4 | 2:21 | 3 | 3 | offscreen | good | 3 pulses corresponding to 3 seal head bobs, seal close to hydrophone | S14 |
| 5/15/2023 | 23 | 3:17 | 10 | 10 | delayed | good | 10 pulses corresponding to 10 seal head bobs | S15 |
